# Exploring Usability Issues of a Smartphone-Based Physician-to-Physician Teleconsultation App in an Orthopedic Clinic: Mixed Methods Study

**DOI:** 10.2196/31130

**Published:** 2021-12-20

**Authors:** Songphan Choemprayong, Chris Charoenlap, Krerk Piromsopa

**Affiliations:** 1 Department of Library Science Faculty of Arts Chulalongkorn University Bangkok Thailand; 2 Behavioral Research and Informatics in Social Science Research Unit Sasin School of Management Chulalongkorn University Bangkok Thailand; 3 Department of Orthopaedic Faculty of Medicine Chulalongkorn University Bangkok Thailand; 4 Department of Computer Engineering Faculty of Engineering Chulalongkorn University Bangkok Thailand

**Keywords:** teleconsultation, remote consultation, mobile applications, usability, orthopedics, physician-to-physician consultation, electronic medical records, mobile phone

## Abstract

**Background:**

Physician-to-physician teleconsultation has increasingly played an essential role in delivering optimum health care services, particularly in orthopedic practice. In this study, the usability of a smartphone app for teleconsultation among orthopedic specialists was investigated to explore issues informing further recommendations for improvement in the following iterations.

**Objective:**

This study aimed to explore usability issues emerging from users’ interactions with MEDIC app, a smartphone-based patient-centered physician-to-physician teleconsultation system.

**Methods:**

Five attending physicians in the Department of Orthopedics in a large medical school in Bangkok, Thailand, were recruited and asked to perform 5 evaluation tasks, namely, group formation, patient registration, clinical data capturing, case record form creation, and teleconsultation. In addition, one expert user was recruited as the control participant. Think aloud was adopted while performing the tasks. Semistructured interviews were conducted after each task and prior to the exit. Quantitative and qualitative measures were used to identify usability issues in 7 domains based on the People At the Centre of Mobile Application Development model: effectiveness, efficiency, satisfaction, learnability, memorability, error, and cognitive load.

**Results:**

Several measures indicate various aspects of usability of the app, including completion rates, time to completion, number of clicks, number of screens, errors, incidents where participants were unable to perform tasks, which had previously been completed, and perceived task difficulty. Major and critical usability issues based on participant feedback were rooted from the limitation of screen size and resolution. Errors in data input (eg, typing errors, miscalculation), action failures, and misinterpretation of data (ie, radiography) were the most critical and common issues found in this study. A few participants did not complete the assigned tasks mostly owing to the navigation design and misreading/misunderstanding icons. However, the novice users were quite positive that they would be able to become familiar with the app in a short period of time.

**Conclusions:**

The usability issues in physician-to-physician teleconsultation systems in smartphones, in general, are derived from the limitations of smartphones and their operating systems. Although some recommendations were devised to handle these usability issues, usability evaluation for additional development should still be further investigated.

## Introduction

Comprehensive personalized care has become a desirable model for health care systems in many countries. Such a model requires the integration of multiple stakeholders and systems to provide patient-centered services. In addition, the efficiency of health care systems, particularly in terms of collecting, storing, analyzing, and accessing patient records and related data is now even more critical to the quality of health care service delivery. Information and communication technologies have advanced health care services in numerous ways, particularly in reducing medical errors, paper consumption, physical storage space, and time. Electronic medical records (EMRs), for instance, have been widely adopted since they play an essential role in the data repository as well as a point of reference in communication between health care providers and patients. Although EMRs are concerned with how health care providers manage patient records, the modern health care system requires collaboration between health care providers, in the expectation of improving the quality of diagnosis and the treatment process, thereby ensuring the quality of data and increasing trust among providers as well as between providers and patients [1].

Teleconsultation is broadly defined and used to explain the remote communication between at least 2 parties in conducting the health care process and services (eg, between a primary care physician and a specialist, between a physician and a nurse, between a resident and a supervising physician, and between a physician/nurse and a patient) [1]. Owing to the advancement of information and communication technology, teleconsultation can be delivered via various channels, for instance, telephone [1,2], video conference systems [3], instant messengers [4-6], and smartphone apps [7]. In particular, the use of mobile devices has increased worldwide since the first introduction of smartphones in the late 2000s. The International Telecommunication Union [8] estimates that there were almost 8 billion mobile cellular subscriptions worldwide in 2020. In Thailand, there were in excess of approximately 119 million mobile subscriptions in 2020 [9]. Mobile phones have become a major platform, bypassing desktops and websites in many other areas.

Teleconsultation has become more common in orthopedic care [10,11], particularly in terms of telemonitoring, teleradiography, and telesurgery [12]. The COVID-19 pandemic has accelerated the adoption of telemedicine, despite criticism and resistance in certain specialties [10,13,14]. There have been several evaluation studies on the effectiveness of teleconsultation in terms of data quality and clinical outcomes (eg, length of stay, user satisfaction, economic evaluation [10,15]). Although the results from systematic review studies cannot confirm the clinical benefits of teleconsultation [15], it is apparent that orthopedic specialists, particularly surgeons, prefer teleconsultation over traditional office visits with patients [10].

Considering that telemedicine and collaborative practice have been increasingly adopted in orthopedic care, a patient-focused teleconsultation platform allows specialists and health care providers to be able to access up-to-date patient records anytime and anywhere. Where diagnosis and prescription are needed, health care providers can update patient records and provide consultation on the go. However, developing a mobile app, in general, can have numerous usability challenges. For instance, limited screen size and resolution restrict the capacity to display large-scale information. Moreover, screen size and resolution may affect the performance of data input, particularly when typing and selecting from a list [16]. Other factors that can affect the usability of mobile apps include distractions during use, connection speed, and processing power [17]. Designers and developers of mobile apps and websites may have to compromise their design in numerous ways, for example, by segmenting and presenting information in multiple pages. Although the usability of mobile apps has been widely studied [17-20], their usability in physician-to-physician teleconsultation has seldom been investigated [7,21,22]. Abundant usability evaluations in health care systems focus on EMRs in the desktop environment [23-25]. Even in the usability studies of mobile EMR systems [26-32], most of them tend to apply generic frameworks rather than those developed for mobile app or teleconsultation specifically. Kim et al [33] called for further explorations of the feasibility, particularly from a usability perspective, of mobile apps on smartphones among physicians.

Harrison et al [18] point out that most mobile usability models focus only on 3 basic attributes, that is, effectiveness, efficiency, and satisfaction, overlooking other essential attributes such as cognitive load. People At the Centre of Mobile Application Development (PACMAD), an evaluation framework, was developed and tailored to address the usability of mobile apps. Based on the International Organization of Standardization and the famous Nielsen’s model [34], PACMAD covers 7 relevant usability attributes, that is, learnability, efficiency, effectiveness, errors, memorability, satisfaction, and cognitive load. Although the constructs in this model cover a wide range of usability aspects and the model has been widely used in various contexts of use, goals, and groups of users, including patient-based mobile health apps [35-37], its application in physician-to-physician consultation mobile apps is very limited. As the framework is applicable to the usability of mobile apps in general, this study adopts PACMAD as the theoretical and analytical framework.

Using a heuristic evaluation approach [38], this study aimed to explore usability issues emerging from interaction with a patient-oriented physician-to-physician teleconsultation app on a smartphone device. Although the app developed can be applied to other settings, this study uses an orthopedic clinic as a setting to control the complexity of the task and the potential confounding determinants [23]. In addition to informing the recommendations and guidelines for the design and development of medical apps on small mobile devices, this study also sheds some light on the feasibility of teleconsultation apps among physicians on smartphones, which are more pervasive and portable. Further, this study investigates the applicability of PACMAD, a general usability framework of mobile devices, in the context of physician-to-physician teleconsultation.

## Methods

### Participants

Nielsen [39] argues that, in exploring usability issues among homogenous users, the first 3 users will help discover problems in an exponential manner. The data are hypothetically saturated after the fifth user. In addition, the primary users of the current version of MEDIC are orthopedic specialists and physicians who normally provide consultations with each other. As a single-site study, the study site was an orthopedic clinic in a large medical school in Bangkok, Thailand, housing around 60 orthopedic specialists and physicians. The study enrolment was announced in the department meeting and all participants joined voluntarily. One orthopedist who regularly used the MEDIC app was recruited to be the control participant. Four specialists and 1 resident who had none or a few experiences of using MEDIC were recruited in this study. To control the complexity of the task and the variability of the platform, the usability tests were conducted using the MEDIC iPhone operating system platform only. Therefore, all participants had to be current iPhone users. The study protocol (IRB 756/62) was approved by the Institutional Review Board, Faculty of Medicine, Chulalongkorn University.

### Data Collection

**Figure 1:**
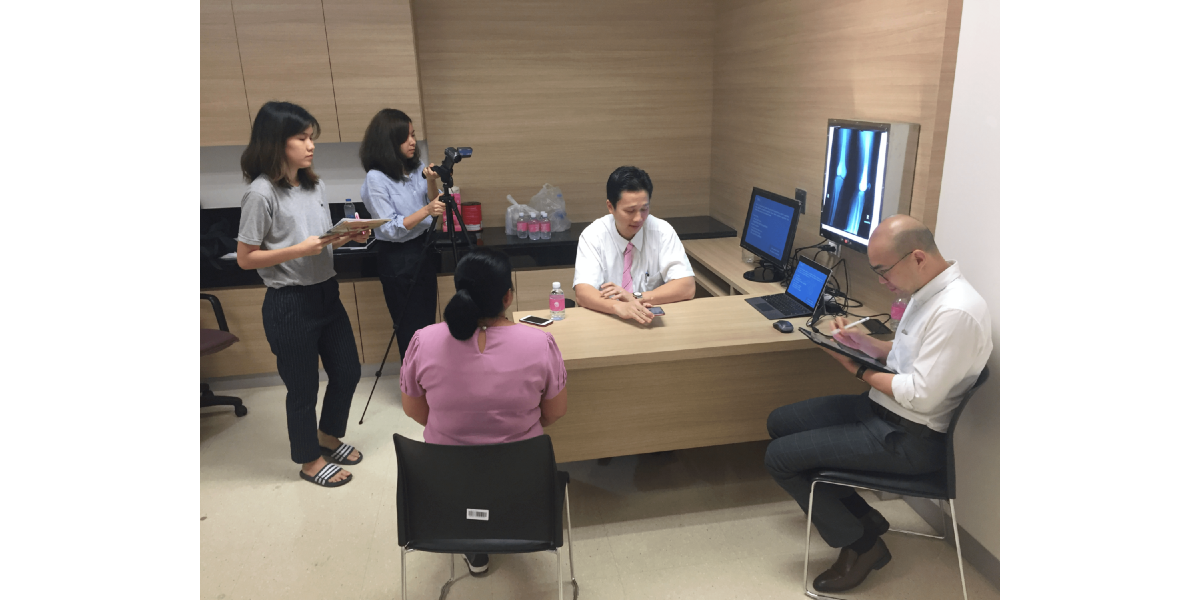
The camera setup for observing research participant interactions with the MEDIC app, while doing history taking and examination with the simulated patient in task 3.

This study was conducted in a controlled setting in the Clinical Skill and Simulation Center (Figure 1). Upon arrival, participants were introduced to the MEDIC app through a 5-minute video presentation providing information on the main features and functions of the app. Thereafter, participants were asked to complete a usability test session. The overall test for each participant took about 90 minutes to complete. There were 5 tasks given to the participants to complete individually. In the context of an orthopedic clinic, these tasks were designed to cover the basic functions of the app and the data capturing process in simulated clinical situations. The participants were required to use the app on the provided iPhone 8. They were also allowed the use of other apps on the device to complete the tasks. Owing to the collaborative nature of the teleconsultation work, the users had to create a private group serving as a sharing space to communicate between physicians. The group could be utilized for a clinical unit, a discussion about a specific case or a group of cases, a research project, or a certain task force. Thus, the first task assigned the study participants to create a group and invite other users to join the newly created group. Since MEDIC is designed to be a patient-focused teleconsultation app, MEDIC allows physicians to create and manage patient records within a group space. Only physicians in the group can view and manage patient records in the group. Therefore, the participants were asked to create a new patient record in task 2. In addition, to evaluate how physicians use the app to manage patient records in an environment similar to the natural setting, the study participants were asked to collect patient information by using the app in a simulated clinical visit in task 3.

Another key function of MEDIC is to allow physicians to communicate in a standardized manner via the data collection form. There are several scales and measures that are essential for the clinical management of patients. Some are standardized, while some are tailored and customized within a group. The data collection form function allows physicians to create and customize a form to be used among the physicians in the group. Therefore, task 4 was designed to investigate how the participants used the data collection form by asking them to create a form to collect a Mangled Extremity Severity Score, which is one of the most common scales used in orthopedic clinical practices. Although the first 4 tasks aimed to observe how the participants created and managed data within the app from a sender perspective (eg, medical students, resident physicians, referrers), the last task was designed to evaluate the usability of the app from the perspective of the receiver (eg, peers, advisors, supervisors, referees). The participants were asked to review existing patients’ records who were asked for consultation. The participants were asked to provide clinical opinions as well as to enter a diagnosis and treatment plan in the consulting case’s record. While performing the tasks, participants were asked to speak aloud their thoughts to the researchers. Participant activities were logged using the video camera and screen recording function of the iPhone. The description of all the tasks is shown in Textbox 1.

**Figure 2:**
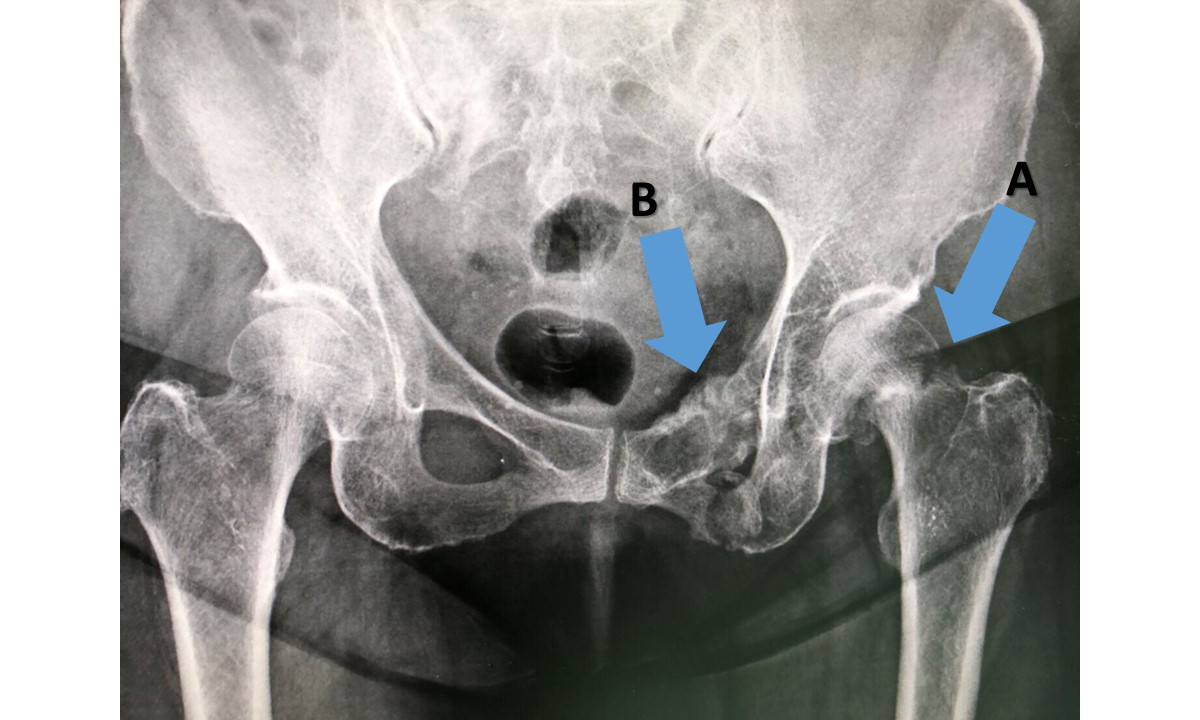
Plain radiograph image, which is used in task 5, showing fractured neck of left hip (femur) (A) and pelvis (pubic rami) (B).

Although there was no limitation on the completion time, participants could leave tasks incomplete at any point in the task. The radiograph used in task 5 is shown in Figure 2. After completing each task, the participants were interviewed using a semistructured interview approach to obtain detailed information regarding their behavior and experience. Additionally, an exit interview was also conducted after all the tasks had been completed. All user actions were recorded using a screen capturing app and video recording. One of the research team members also observed the participants and recorded their actions in an observational form.

**Tasks in the usability test of the smartphone-based physician-to-physician teleconsultation app.**

**Task 1:** Creating a group, then adding team members and form into the group

**Task 2:** Registering the patient into the group

**Task 3:** Using MEDIC app during a clinical encounter with a simulated patient, including the recording of a radiograph, a photo of the affected body part (the knee), a physical examination, diagnosis, and plan management. The simulated patient was informed about the case and trained by the researcher. The scenario for task 3 was a 42-year-old female patient presenting with chronic pain in her right knee for 3 months. A plain radiograph showed that she had osteoarthritis, which is a common degenerative condition of the knee joint. The treatment included medication, physical therapy, and surgery.

**Task 4:** Create a new record form, a Mangled Extremity Severity Score, which is used to assess limb-salvage potential of traumatic extremity. Prior to this task, participants were given a brief video introduction on how to create the form.

**Task 5:** Using MEDIC for a teleconsultation of orthopedic trauma cases. Participants were asked to review a patient’s radiograph and then provide a diagnosis and treatment plan as well as clinical opinion. The task 5 case was a 35-year-old female who had had a traffic accident 2 hours before arrival at the emergency room. A plain radiograph showed a fractured neck of the left femur, which is the proximal part of the thigh bone, and pubic rami fracture of the pelvic bone.

### Data Analysis

The data from the think-aloud protocol and the semistructured interviews were transcribed. The observation notes were validated with the video recording. Quantitative data, for instance, time, number of clicks, and number of screens used, were recorded in MS Excel and analyzed using descriptive statistics by comparing with expert users’ performance. Thematic analysis using a deductive coding approach was adopted to analyze the transcripts, observation notes, and screen and video recordings by using the qualitative data analysis software, NVivo version 12 (QSR International). The primary coding scheme adopted PACMAD usability attributes [18], including effectiveness, efficiency, satisfaction, learnability, memorability, error, and cognitive load. The coding was conducted by 2 assessors independently. The codes and categories were then compared. Any disagreements were resolved by discussion between the 2 assessors.

### Evaluation Measures

To explore the usability issues of MEDIC, Table 1 shows the measures collected during and after tasks. To triangulate the results, the data were derived from 3 data sources: observation, think-aloud responses, and interviews.

It is apparent that certain measures were attributed to more than one usability domain, for instance, perceived task difficulty addresses both learnability and cognitive load. The time used to accomplish assigned tasks was also used to evaluate learnability and efficiency. For cognitive load, although the NASA Task Load Index is normally recommended [18,40], we considered Flood’s hypothetical approach [41] instead since it specifically addresses the cognitive load in a mobile environment in the context of clinical and health care practices. Furthermore, 2 additional measures were collected to understand user characteristics: (1) familiarity with each task assigned (rating on 1-7 Likert scale) and (2) familiarity with heavy-loaded tasks (eg, writing, filling out a form) on mobile platforms.

**Table 1:** Measures of usability.

Usability attributes	Data sources		
	Observations during task performances	Posttask interviews	Exit interviews
Effectiveness	Completion rate using the Laplace method Comparing the time to completion, number of clicks and number of screens with expert performance	—^a^	—
Efficiency	Time to completion Number of clicks Number of screens used	—	—
Satisfaction	—	—	Perceived potential impact of the app on the effectiveness of current workflow
Learnability	Time to completion	Perceived task difficulty	—
Memorability	—	Number of incidents where participants were unable to perform a task, which had previously been completed	—
Error	Incidents when errors occurred	—	—
Cognitive load	Distractions during task performance	Perceived task difficulty	—

^a^Not available.

### MEDIC: Smartphone Physician-to-Physician Teleconsultation App

MEDIC, developed by Deverhood, Thailand, is a smartphone teleconsultation app for physicians to communicate with each other in various settings. As the app is designed to support patient-centered care, the main features of the app include patient medical data such as medical history, physical examination, clinical images, and diagnostic questionnaires. Health care providers can access, collect, and modify data from both desktop and mobile platforms, including both iPhone operating system and Android. However, the testing version in this study was on the iPhone operating system platform to control the environment. All data were to be uploaded to the cloud server; therefore, an internet connection was required while using the app. Designed to support collaboration among physicians and specialists, the MEDIC interface is divided to support 4 main tasks, namely, forming a team, data form creation, data recording, and data reviewing. The first task begins with forming a team such as a research group or a multicenter collaboration by creating a group and adding members. To invite members to the group, all teammates must have accounts with MEDIC. Group members can be removed or included by the group administrator.

**Figure 3:**
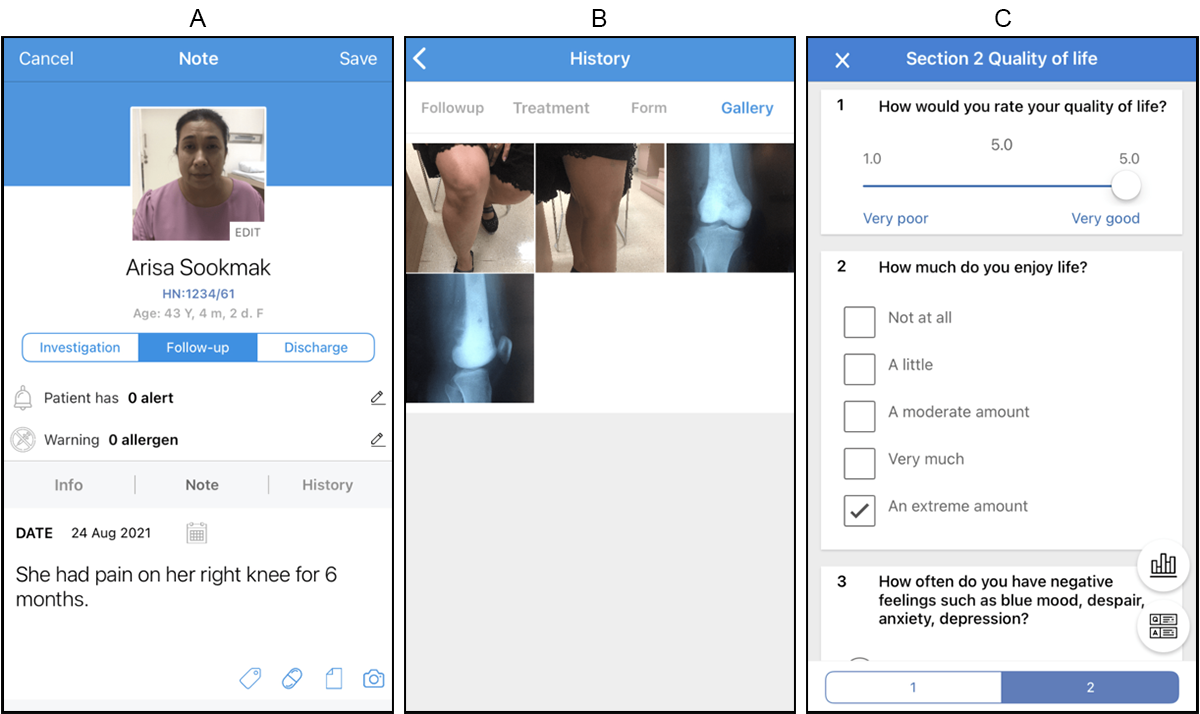
Three screenshots of the MEDIC app: patient history note (A), image gallery (B), and record form (C).

Although MEDIC has been designed to collect generic patient records (eg, demographic, diagnosis, medical history, treatment), the app also allows physicians to create a data form to support their specialty, such as a case record form and functional score. However, licensed questionnaires should be authorized by the licensed owner in advance. There are 10 data input formats that can be used in the form: check boxes, drop-down lists, multiple choice, linear scale, multiple choice grids, free text, number text, date-time, picture, and video link. The content of the form can be organized into a section. Each question can be set as a required status, which should be completed or the form cannot be submitted. Relevant forms should be assigned to the related group. The data capturing process begins with patient registration with the group by entering a general profile and collecting data using a free text box, form, and camera tool. All recorded data can be reviewed by pressing the previous history tab, which shows free text history, forms, and images that were recorded in the past. Apart from all the main features, this app allows users to fill out their profiles for reference and a setup passcode lock to increase data security. The main features of the app are shown in Figure 3.

Apart from the capability of clinical data capturing, MEDIC is suitable for teleconsultation between health care personnel. The MEDIC app provides organized information, including history, laboratory findings, and clinical and radiological images, and patient condition and management are automatically sorted in a chronological order so that it is convenient for reviewing disease progression and treatment plan. Patient data privacy protection is improved by using MEDIC instead of social networks such as WhatsApp or Facebook messenger because the data access is limited to authorized persons for use in patient management.

## Results

### Participants’ Characteristics

#### Demographics

All participants were males whose age ranged from the late 20s to early 40s. One of the participants was in the final year of orthopedic resident training while the others were board-certified orthopedists. All participants, reportedly, were highly familiar with and had been using smartphones for many years. One expert was an orthopedist who used the MEDIC app regularly and had been involved with the development of the app.

#### Familiarity With Usability Tasks

The participants were asked to declare how familiar they were with each given task. As shown in Table 2, they tended to be the most familiar with task 2 and task 4 (median=6). For the other tasks, familiarity was distributed among the 5 participants.

We also asked the participants to provide feedback on their familiarity with heavy-loaded tasks on mobile platforms. All participants said they were familiar with multitasking on smartphones with regard to work-related tasks.

**Table 2:** Familiarity with tasks (N=5).

Task	Median (range)
1	3 (1-7)
2	6 (4-7)
3	3 (1-5.5)
4	6 (4-6.5)
5	4 (1-7)

### Usability of the MEDIC App

The following section reports different measures covering all 7 usability dimensions of PACMAD in the smartphone-based physician-to-physician teleconsultation app, that is, completion rates, time to completion, number of clicks, number of screens, errors, incidents where the participant was unable to perform a task, which had previously been completed, and perceived task difficulty.

#### Completion Rates

As shown in Table 3, all participants completed tasks 2, 3, and 4 (completion rate=100%; Laplace=0.86). One participant did not complete task 1 and 5 (completion rate=80%; Laplace=0.71). One participant did not complete task 1 because he failed to add a user (another physician) into the created group. For task 5, the participant could not locate or contact another physician for consultation for a specific case.

**Table 3:** Completion rates (N=5).

Task	Completion rate, n (%)	Laplace
1	4 (80)	0.7143
2	5 (100)	0.8571
3	5 (100)	0.8571
4	5 (100)	0.8571
5	4 (80)	0.7143

#### Time to Completion (Minutes)

As shown in Table 4, the participants completed task 1 taking about 3 times longer (median=6 minutes) than the time used by the expert (median=2 minutes). For task 2, the median time used by participants (median=2 minutes) was about the same as the time used by the expert. For task 3, the participants used about 16 minutes to complete the task, while the expert used about 6 minutes. It is noteworthy that task 3 involved interviewing a simulated patient. Therefore, the range of time used was from 8 minutes to 24 minutes. The participants completed task 4 using about 8 minutes, approximately 1.25 times more than the time used by the expert. The median time spent by the participants was approximately 2 times more than that spent by one of the experts (6 minutes and 3 minutes, respectively).

**Table 4:** Time (minutes) used by given tasks (N=5).

Task	Median (range)	Expert
1	6 (2-8)	2
2	2 (1-6)	2
3	16 (8-24)	6
4	8 (6-11)	6
5	6 (4-8)	3

#### Number of Clicks

We also observed the number of mouse clicks during each task, as illustrated in Table 5. For all tasks, except task 2, the median number of clicks by the participants was higher than those by the expert (41 versus 27 in task 1, 88 versus 27 in task 3, 92 versus 68 in task 4, and 36 versus 18 in task 5). The greatest difference between the median number of clicks by participants and the number of clicks by the expert was in task 3 (about 3.26 times higher). However, there was 1 participant who could complete task 3 within 17 clicks, which was lower than the number of clicks by the expert. For task 2, all participants completed the task by using a lower number of clicks than that used by the expert (13 and 19, respectively).

**Table 5:** Number of clicks by given tasks (N=5).

Task	Median (range)	Expert
1	41 (15-41)	27
2	13 (10-14)	19
3	88 (17-133)	27
4	92 (74-131)	68
5	36 (28-45)	18

#### Number of Screens Used

In terms of screens used, as shown in Table 6, the median number of screens used in all tasks was higher than that of the screens used by the expert. For task 1, the median number of screens used was 25 screens, while the expert used 17 screens. The participants completed task 2 by using 6 screens (median) compared to 4 screens by the expert. The median number of screens used by the participants in task 3 was much higher than that used by the expert (46 and 17, respectively). In task 4, the median number of screens used by the participants was 23 screens, while the expert used only 17 screens to complete this task. The participants completed task 5 by using 31 screens (median) compared to 12 screens used by the expert. It is also noteworthy that some participants used fewer screens to complete tasks 1, 3, and 4, compared to the number of screens used by the expert.

**Table 6:** Number of screens visited by given tasks (N=5).

Task	Median (range)	Expert
1	25 (10-40)	17
2	6 (4-7)	4
3	46 (9-72)	17
4	23 (14-29)	17
5	31 (16-38)	12

#### Errors

In this study, we observed errors through direct observation as well as through user feedback. Three types of errors were identified: action failure, data inaccuracy, and error recovery failure.

The first type of error was action failure. The participants could not complete certain activities. For example, in task 3, one participant entered the diagnosis information into a wrong section. It is apparent that the term “form” was used in multiple sections where it was meant differently depending on in which section it appeared. Therefore, this discrepancy led to subsequent confusion and data entry error. Another major action failure dealt with navigation issues. Some participants could not navigate the app or correctly locate the section where they expected to complete tasks. Apparently, they did not understand the vocabulary and icons used. In addition, in task 1, where they were asked to create a group and add a member into the group, most participants took a lot of time navigating through the app to add a member into a created group. Most of them used a trial-and-error approach by browsing and clicking all buttons to see if they were helpful.

The action failure was found to be related to data inaccuracy, which is another type of error found in this study. In task 4, where they were asked to create and complete an assessment form, 3 participants entered data into incorrect fields because they were confused about how to create and fill out the information in the form. In addition, 1 participant incorrectly input the patient birth year from 1978 to 1987. This incident was led by the discrepancy between the calendar year system required in the app (Gregorian calendar year) and the official local calendar system (Buddhist calendar year). The participant had to manually add an extra step to convert the difference between the two calendar year systems, which increases the risk of data inaccuracy.

Another error was the frustration to recover after encountering an error. There were a few incidents where the participants found certain mistakes where they would have liked to make some changes. However, they could not find a solution to this for 2 main reasons. First, the app did not have an edit function for specific tasks. Second, the participants’ mental model about how to edit did not match the edit operation in the app. For instance, participant 2 tried to create a form to collect patient data in task 4. However, after adding a question, the participant found that he had misplaced the order of the question. He struggled to find a workaround to reorder the question. He ended up deleting the entire questionnaire and started creating a new form instead.

#### Incidents Where a Participant Was Unable to Perform a Task That Had Previously Been Completed

Based on a direct observation during the tests and validated by 2 observers, there was no incident where participants were unable to perform a task, which had previously been completed.

#### Perceived Task Difficulty

To evaluate the cognitive load during task performance, we asked the participants to rate perceived task difficulty on a 7-point Likert scale (1, not difficult at all; 7, most difficult). Based on the medians, as shown in Table 7, the perceived difficulties in all tasks were considered as moderate to most difficult depending upon each task assignment. However, considering the range, some participants rated tasks 1, 3, and 5 as less difficult.

**Table 7:** Perceived task difficulty rating (N=5).

Task	Median (range)
1	3 (1-7)
2	6 (4-7)
3	3 (1-7)
4	6 (4-5.5)
5	4 (1-5.5)

Moreover, we asked the participants to provide feedback supporting their ratings. We analyzed their feedback in relation to cognitive load. For those who rated tasks as less difficult, the rationale supporting their perception included the clarity of the interface, compatibility with their workflow, and the familiarity of the task to current practices (eg, creating a group and adding a group member). In addition to the perceived task difficulty, we also observed the distractions during task completion in all tasks. However, those who perceived given tasks as difficult provided feedback that certain actions required additional resources (eg, time, memory). Participant 2 commented on the difficulty of converting the date of birth from the Buddhist calendar year to the Gregorian calendar.

…Well, for entering the date [of birth], sometimes, I need time to think and to fill in the information. It takes time to do so. But we really don’t have a lot of time for each patient.

Participant 2

For some participants, typing on a small screen was another intensive task. Participants reported some difficulty in typing on the screen. One participant said that his fingers were too big for the screen, causing misalignment with the keyboard. Participant 3 compared typing with taking a photo of a handwriting chart. He felt that writing on paper and taking pictures was faster than typing. Especially when typing while meeting patients, the participants felt that they were distracted by how much attention was required to focus on what was typed rather than the interaction with the patient. To compromise the cognitive load during typing, participant 3 decided to keep the notes concise, for instance, by using abbreviations and short phrases. It is noteworthy that omitting certain information in the medical record can cause cognitive load in recalling the information. For example, time-sensitive information requires a specific unit. Participant 2 expressed his frustration regarding the ambiguity caused by lack of contextual information.

…For the question ‘how long ago did the patient have surgery? I usually put the unit, like month, in the chart. If it only has a number, I have no idea what this number means. For example, if I see number 6, what does it mean? 6 months? 6 days? or 6 years? I cannot tell.... It makes it a bit difficult to communicate with the patient.

Participant 2

Another incident concerned unfamiliarity with the form creation process in task 5. MEDIC allows physicians to create a customized form to collect certain information for further evaluation. For those who have never created a form before, they were confused by the terms used in the app, for example, section, question, dropdown menu, and check box. They spent a great amount of time trying to figure out how to create a form.

Another task that was rated difficult in some responses was form creation in task 4. A number of participants were unfamiliar with the vocabulary and the process of form creation. For example, the app allows the users to separate a questionnaire into multiple sections. However, the assigned scale, Mangled Extremity Severity Score, contains 4 questions, which do not require subsections. A couple of participants were confused about the term “section” in the form creation function. They took some time to understand the difference between the term section and question. This is perhaps partly because the items of the assigned scale are not presented in a question statement but rather in a heading format (eg, skeletal/soft-tissue injury, limb ischemia, shock, age), which could be assimilated with section titles, rather than questions.

A camera can be a useful function to capture patient records. MEDIC also provides an in-app camera function so that the user can embed photos related to patient records. The in-app camera can save time and cognitive load. However, during the test of task 3, one participant used a mobile camera instead of the in-app camera. Although the participant was able to finish the task assignment, it took him extra time and effort to completely locate the photos as well as upload them into the app.

#### Potential Impact of Using the App on the Current Workflow

To evaluate user satisfaction with the app, we asked all participants their opinion about the potential impact of using the app on the current workflow. All participants tended to have a positive attitude toward the impact of using the app in their practice. They thought it would likely improve the efficiency of the current workflow. The app was preferred to using instant messaging apps to communicate among physicians regarding a patient’s prognosis and treatment plan.

...I think it is convenient to use for consultation across departments, especially for cases that need long-term care and continual discussions. It seemed impractical to use [an instant messenger app]. The problem is the chat room contains discussion and records of multiple cases. So, we have to find relevant information from a very long conversation. It is much better to get a whole patient record at once.

Participant 1

Nevertheless, a few participants (n=3) addressed the point that familiarity with the app would be the most substantial condition that affects the efficiency of the workflow. In addition, the performance of the app was also another factor raised by a couple of participants.

...Firstly, I think it’s about familiarity with the tool. If you don’t use it every day, you won’t get used to it. Secondly, it depends on the app itself. The issues related to the app, for example, misalignment of the interface and delayed or frozen app, forced me to quit and/or restart it. Anyway, the point is that if you aren’t familiar with it, it will always be difficult to use.

Participant 2

## Discussion

### Feasibility of the MEDIC App Adoption

This study aimed to discover usability issues related to the smartphone-based physician-to-physician teleconsultation system, MEDIC, by using a mixed methods approach. From the summative evaluation perspective, MEDIC seems to be promisingly satisfactory as an app providing opportunities for efficient communication among health care providers, improvement of privacy protection, and increasing accessibility to patient records outside the clinic as well as in the context of long-term care. In addition, MEDIC is designed and perceived as a patient-centric platform where all related records and documents are organized for each patient. The participants generally compared MEDIC with their current practices in recording patients records and consulting with other health care providers (ie, paper chart, EMR system, and instant messengers). Using instant messengers can be frustrating when discussing multiple issues and cases in one channel. One participant reported that he normally writes patient records on paper and takes photos of the paper using his own smartphone. Although it can be convenient for capturing data, the quality of the photos of the records can be poor owing to the capturing process. In addition, it takes some time to retrieve the photos since such a process greatly relies on recall and memory. The current EMR system at the clinic is not portable and is not flexible in terms of serving specific needs and medical practice.

### Usability Issues Related to Mismatched Mental Models

The results of participants’ performance (ie, number of screens used, number of clicks, time used) in relation to the experts’ performance can be considered as acceptable, considering that the majority of them had never used the app before. For memorability, the results yield a positive perspective since none of the participants forgot any actions that they had completed earlier during the test. However, usability issues were the most visible in learnability, errors, and cognitive load. Qualitative data were very helpful to explore users’ mental models in addressing these issues. Although these issues emerged from the interaction with MEDIC, most of the feedback can be applied to smartphone-based teleconsultation systems in general. The most frustrating task for some participants was creating a patient evaluation form (task 4) based on the number of clicks and time spent as well as the comments during and after completing the task. Although all of them were familiar with the assigned scale, that is, Mangled Extremity Severity Score, the participants who were not familiar with the vocabulary related to electronic form and questionnaire development (eg, dropdown, checkbox, select option) found it difficult to understand the interface for the first time. They took a longer time to complete it since they applied a trial-and-error approach to become familiar with the form creation process. Nevertheless, they commented that it would take them only a couple of hours to get familiar with this task. Creating a usable data collection form (eg, measurement scales, questionnaires) has been reportedly one of the most challenging tasks in system design and development from a broad perspective [42,43]. In a clinical context, numerous established measurements have been extensively used to assist the delivery of health care services. Creating a data collection form in a mobile app based on existing paper-based questionnaires can be challenging for novice users since it may require a different mental model [44]. Users may need to be familiar with the available features, icons, and labels to effectively create a form. To address this issue, an introductory guide or a tutorial video about form creation could be useful for users who are using this function for the first time (Multimedia Appendix 1). At the same time, further studies should be conducted to investigate the mental models of novice users particularly on creating an electronic data collection form in health care settings.

### Usability Issues Related to the Screen Size of Smartphones

Another common error among participants was related to the limited image resolution of the smartphone. Neither computer screens nor mobile phones were initially designed to be medical devices. The display size affects the usability performance in multiple ways [45], particularly issues related to data presentation and input. In task 5, we used the image of a pelvic fracture with osteoporosis in a consultation task. It is noteworthy that 4 out of 5 orthopedists failed to recognize pelvic fracture on the screen, even though all participants increased the magnification of the image using the zoom-in function. After this revelation, they commented that they had not seen the fracture or had not paid attention to it because the image was too small. This may reflect the observation that the typical size of mobile phone display may not provide sufficient detail for a radiographic diagnosis [37,46]. In the acute management of multiple bone fractures, underlying conditions such as osteoporosis and other metabolic bone disease should be investigated preoperatively for proper surgical preparation and medical management to prevent unexpected complications. However, Hasselberg et al [15] found that diagnosis validity does not only depend on technology but also the users’ experience and physical ability. Certain solutions were suggested to mediate this issue, for instance, using an integrated DICOM viewer to provide better contrast, an ability to project onto a larger screen, and showing an image scale and other contextual information to raise awareness.

Another related issue as a consequence of screen size limitation is the misinterpretation and confusion about the image icons, which have also been addressed by other studies [7]. Owing to the limitation of screen size and the large amount of data, the design team decided to use image icons in place of text labels extensively in the app, especially for buttons. The decision led to issues related to naturalness, lack of information, and misleading information that are commonly found in other usability studies on smartphone-based health apps [37]. Participants who were using the app for the first time indicated that some of the icons were ambiguous and led to frustration. Some commented that some icons were too similar. When they were not sure what these icons were, they normally clicked to see where the buttons led to. This would cause frustration and be time consuming if the buttons did not lead to where they expected. However, some participants were successful in identifying the icons, referring to contextual elements, such as location of the icons, displaying content, and nearby icons. While replacing all icons with text labels would be immensely challenging, one possible solution is to use hover text, a tooltip text appearing when a user moves the cursor over a button. However, hover text is still not common in a number of developing platforms on touch screen devices. Other recommendations to improve the understandability of icons include removing unrelated or “unnecessary” icons, redesigning the icons to improve the distinctiveness among them, and providing a tutorial guide for the first-time user. These solutions are also suggested in other literature to avoid feature fatigue [47-49].

In addition to data presentation, the low resolution of smartphone screens can lead to data input errors [50-52]. We found that typing and clicking mistakes were omnipresent in all tasks. Participants commented that they usually had typing issues on their smartphones regardless of the app. They commented that the on-screen keyboard is too small. It is important to note that none of the participants used the swipe type function, where a user can glide his/her finger between characters. During task 3, one participant put the smartphone down and jotted down all the information on paper while talking to the simulated patient. He commented that typing on the mobile phone screen was difficult and required a lot of attention. Typing would significantly distract him from having a conversation with the patient. Alternative input methods were also suggested by the participants to remedy this issue, for example, adding an audio recording function and speech recognition ability as well as providing contextualized word suggestions. In addition, conditional formulae, such as deactivating a button/input when it is irrelevant, would help reduce errors in typing and other calculation tasks.

**Figure 4:**
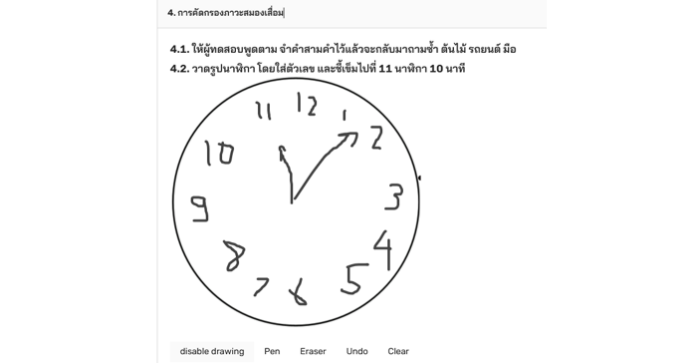
A prototype interface for clock drawing in Montreal Cognitive Assessment on MEDIC app.

Drawing was also another input method recommended by the participants. Although MEDIC allows users to take photos, some participants commented that taking pictures alone might not be enough to capture all the information they would like to add. During the consultation in task 3, one participant mentioned that he wished to annotate the images taken by either drawing or typing next to the area of interest (eg, pain site). In addition, some of the gold-standard diagnostic scales require drawing as an input. For example, the Montreal Cognitive Assessment uses clock drawing to evaluate visuoconstruction skill [53]. Therefore, the next iteration of the app development has added a drawing function as well as image annotation as shown in Figure 4. However, it is important to further investigate the usability of drawing functions on smartphone screens since it has been reported elsewhere regarding user frustration [7].

### Applicability of PACMAD Framework for Usability Evaluation of Smartphone-Based Telemedicine Apps

In this study, the PACMAD framework was applied to guide the usability evaluation of a physician-to-physician teleconsultation app. We believe this is the first study utilizing this framework in this telemedicine context. PACMAD seems to be helpful to explore a broad range of usability issues of mobile apps in line with the heuristic evaluation approach. There is currently no specific usability framework specific to smartphone-based telemedicine apps. Although PACMAD was developed as an evaluation framework tailored to the usability of mobile apps in general [18], medical apps and systems can be more sensitive in certain usability aspects, for example, naturalness, consistency, and error prevention [54]. In addition, the evaluation in medical context should consider the complexity of usability from various perspectives, including user skills, task complexity, data sensitivity, and complicated functionality. Smelcer et al [23] argued that understanding the depth and breadth of user knowledge in context is central to the usability of EMR. There are specific frameworks/guidelines addressing the usability of medical systems, in particular [55,56], such as the TURF (task, user, representation, function) framework [25,40]. Applying these frameworks may have helped explore the complexity of usability issues in medical context; however, these frameworks were developed based on the context of desktop-oriented or web-based EMRs. Some are designed based on systems interacting with patients (eg, personal health records, physician-to-patient teleconsultation). It is apparent that PACMAD is more approachable and applicable to formative evaluation approaches where researchers are less restrained from sophisticated constructs and complicated research design. Even though applying more than one framework may be achievable, we found that both frameworks are not totally compatible. For instance, TURF includes both methodological and constructive guidelines, while PACMAD focuses more on usability dimensions. It would be ideal to develop a particular framework to evaluate the usability of medical apps on a mobile (and perhaps tablet) platform.

### Study Limitations and Recommendations for Future Studies

The main objective of this study was to explore the usability issues of MEDIC. Although Nielsen [39] suggests that 5 users would be sufficient to obtain the majority of issues, we found that a larger number of participants would enhance and increase the reliability of the results, particularly in the quantitative analysis. In addition, a more heterogeneous sample would be sufficient to investigate the variation of usability of EMRs based on different user characteristics found in other studies (for instance, between attending and resident physician [57]). In addition, although MEDIC is designed as a physician-to-physician teleconsultation app, the specifications and configurations of the app are still at the early stage. Furthermore, the tasks were developed in the context of an orthopedic clinic in a large medical school. Therefore, the results from this study may not be generalizable to a larger population. MEDIC is not designed to be used as a comprehensive standalone app but to be used in conjunction with other modes of communication among physicians (eg, instant messaging, EMR systems). The feasibility of an integration between patient-based teleconsultation apps and instant messengers should be further explored with respect to patient privacy and safety. Since it seems that screen size plays an essential role in the usability issues of smartphone apps, further studies should investigate alternative measures to prevent errors in data entry, which is the most visible and concerning issue found in this study. Although the display technology of smartphones has been progressively improved, other modes of data entry such as voice, drawing, and click-and-point may be considered in terms of feasibility and usability.

### Conclusion

Since this study investigates the usability of smartphone-based teleconsultation in the early stage of the iterative design process, the purpose and approach of this usability study is rather exploratory than conclusive. While applying a mixed-method approach to gain a comprehensive perspective across all usability dimensions, based on PACMAD, we found that the qualitative data provided insightful perspectives and helped us discover usability issues in numerous aspects. In addition, since the goal of this study was to explore usability issues and the population was quite homogeneous, the feedback from 5 participants was sufficient to discover usability issues in all usability dimensions. Although there are a number of opportunities to improve communication among health care team members as well as between health care professionals and patients, we found that the usability issues of smartphone-based teleconsultation platforms in this study were mostly concerned with learnability, errors, and cognitive load. We found serious issues regarding errors particularly due to the limitation of screen size and resolution. Such limitations impact on how physicians enter and view patient’s records, which subsequently affect the diagnosis and treatment. Although the limitation of screen size has already been discussed in the literature, this study provides empirical evidence from a practical and user-oriented perspective. As in any early stage of development, there are still numerous opportunities for improvement, particularly regarding usability. An iterative process is planned to be adopted to develop the app to be more usable and expandable to a broader user group.

## Appendix

Multimedia Appendix 1 Tutorial video presentation of the MEDIC app.

## Abbreviations

EMR: electronic medical record
PACMAD: People At the Centre of Mobile Application Development
TURF: task, user, representation, function
