# Supplementary material for: Exploring Usability Issues of a Smartphone-Based Physician-to-Physician Teleconsultation App in an Orthopedic Clinic: Mixed Methods Study
Source: JMIR Hum Factors. 2021 Dec 20;8(4):e31130. doi: 10.2196/31130 (PMC8726029; doi:10.2196/31130)

## Slide 1
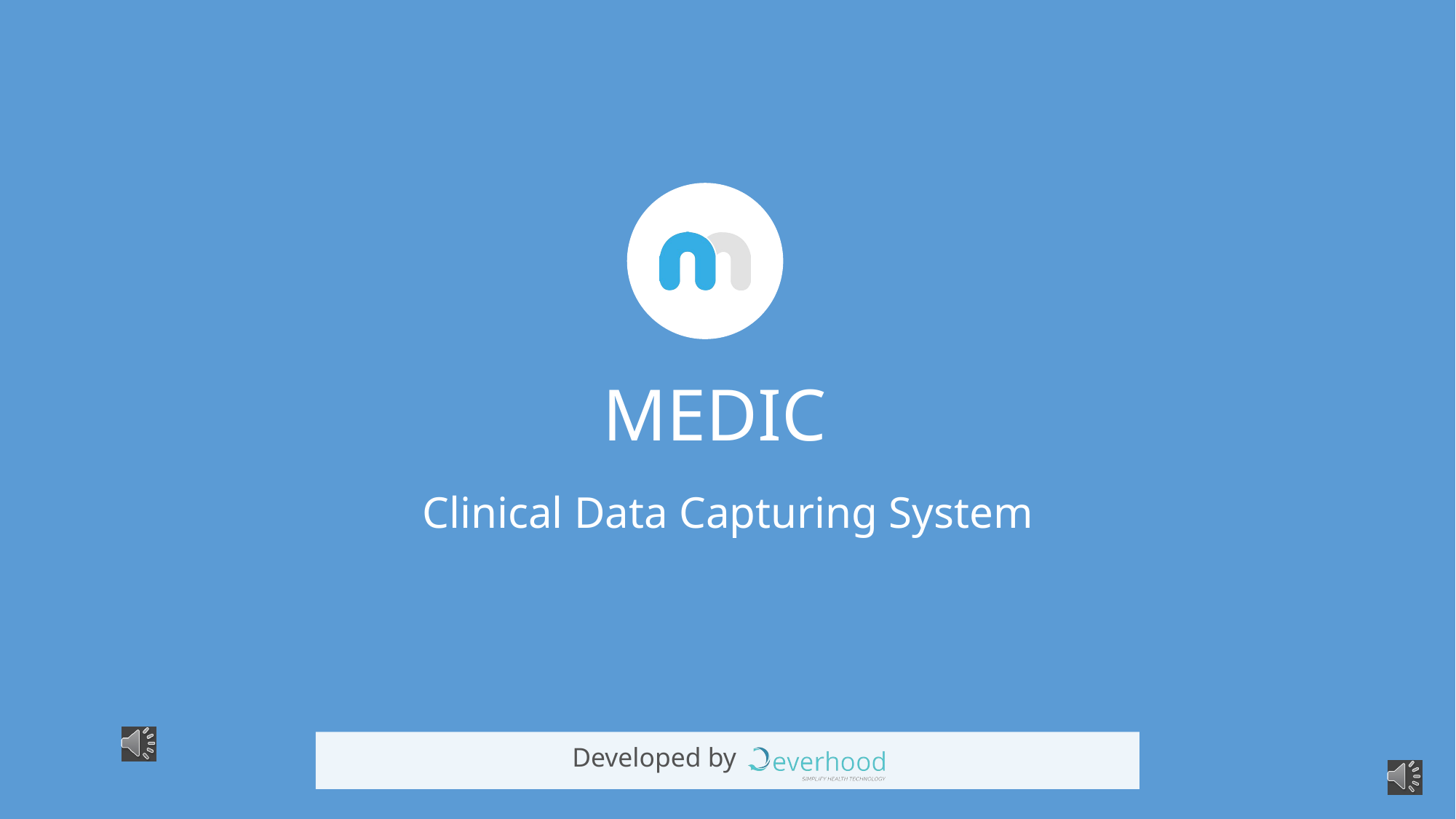

## Slide 2
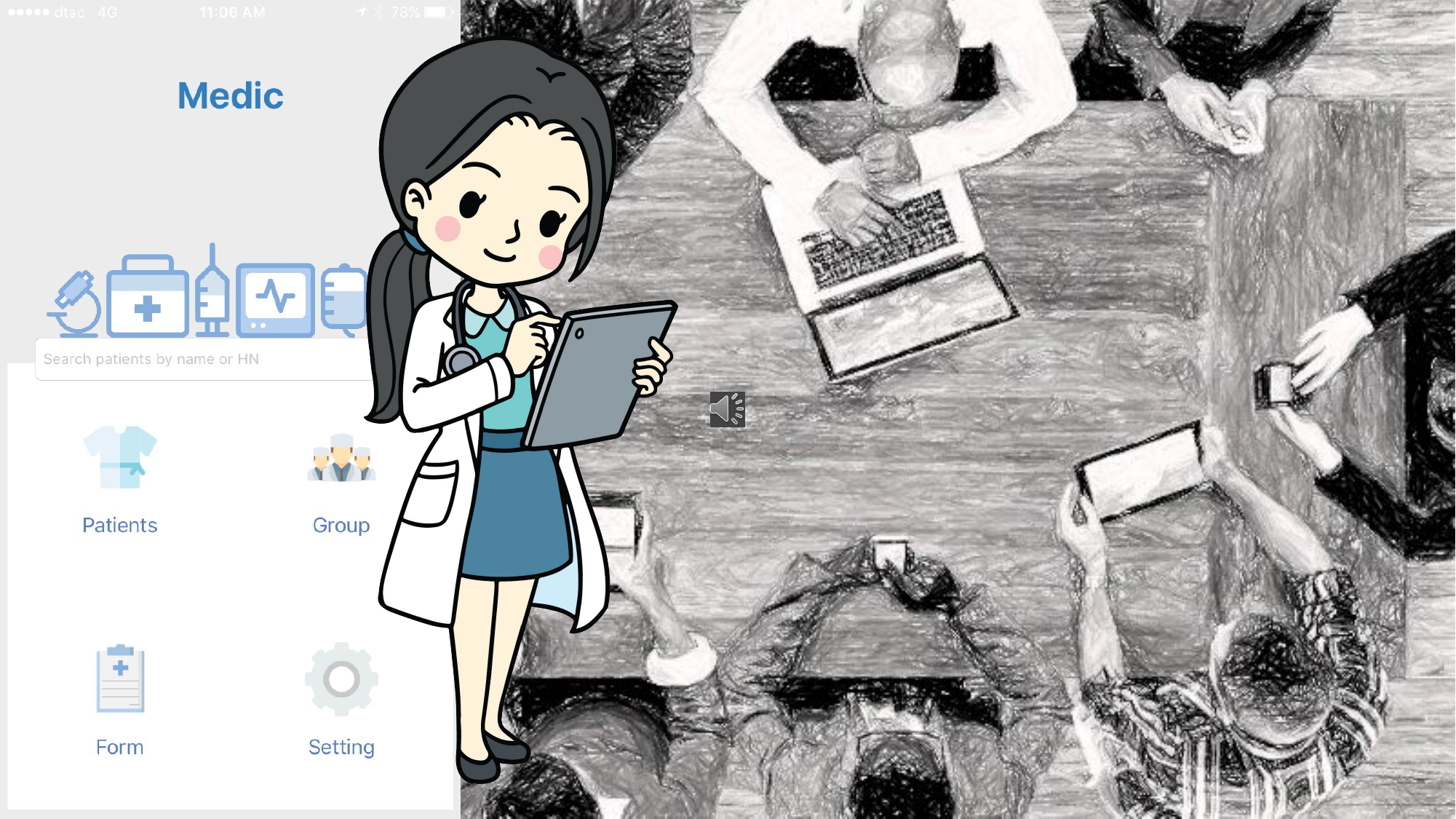

## Slide 3
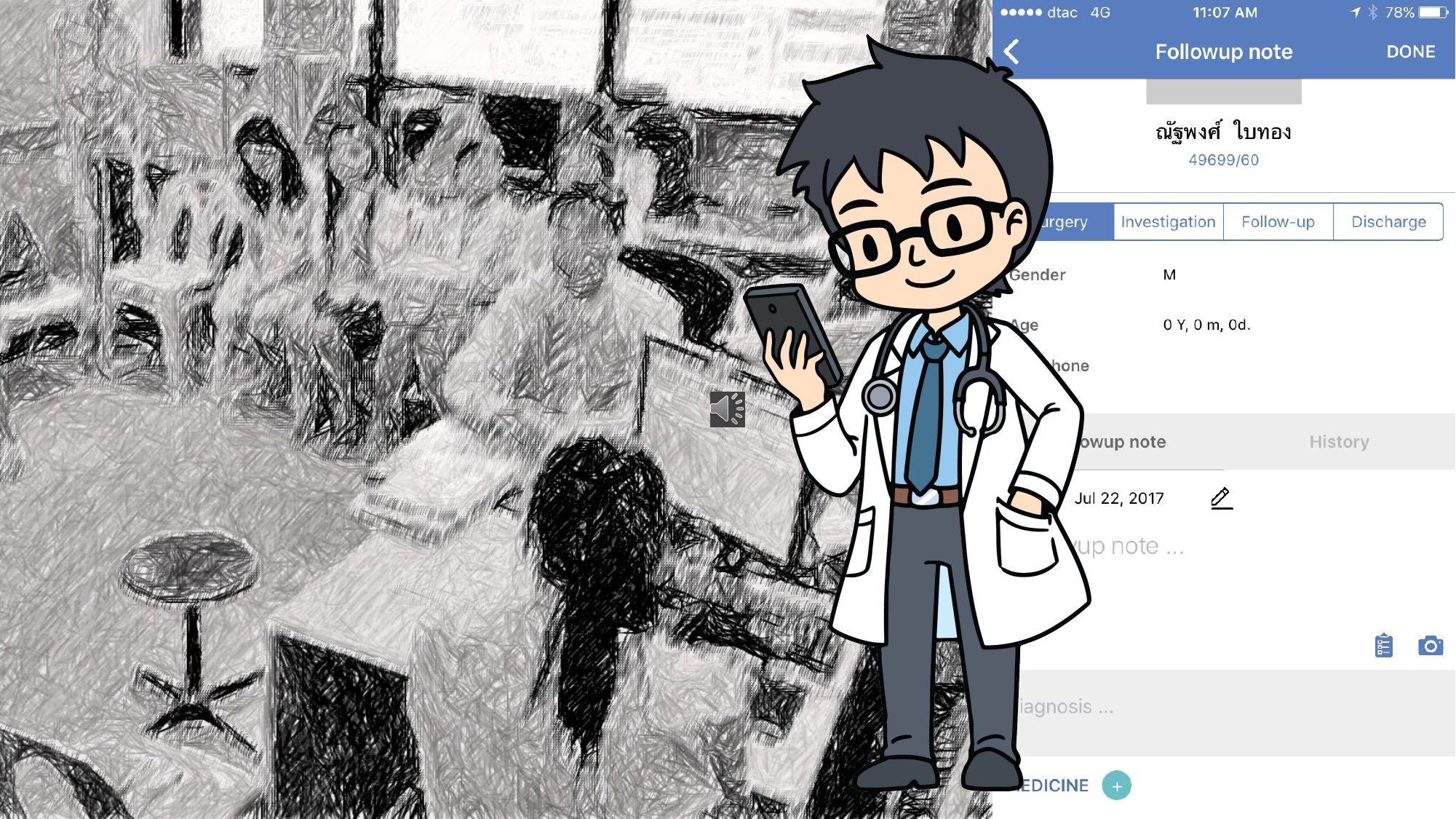

## Slide 4
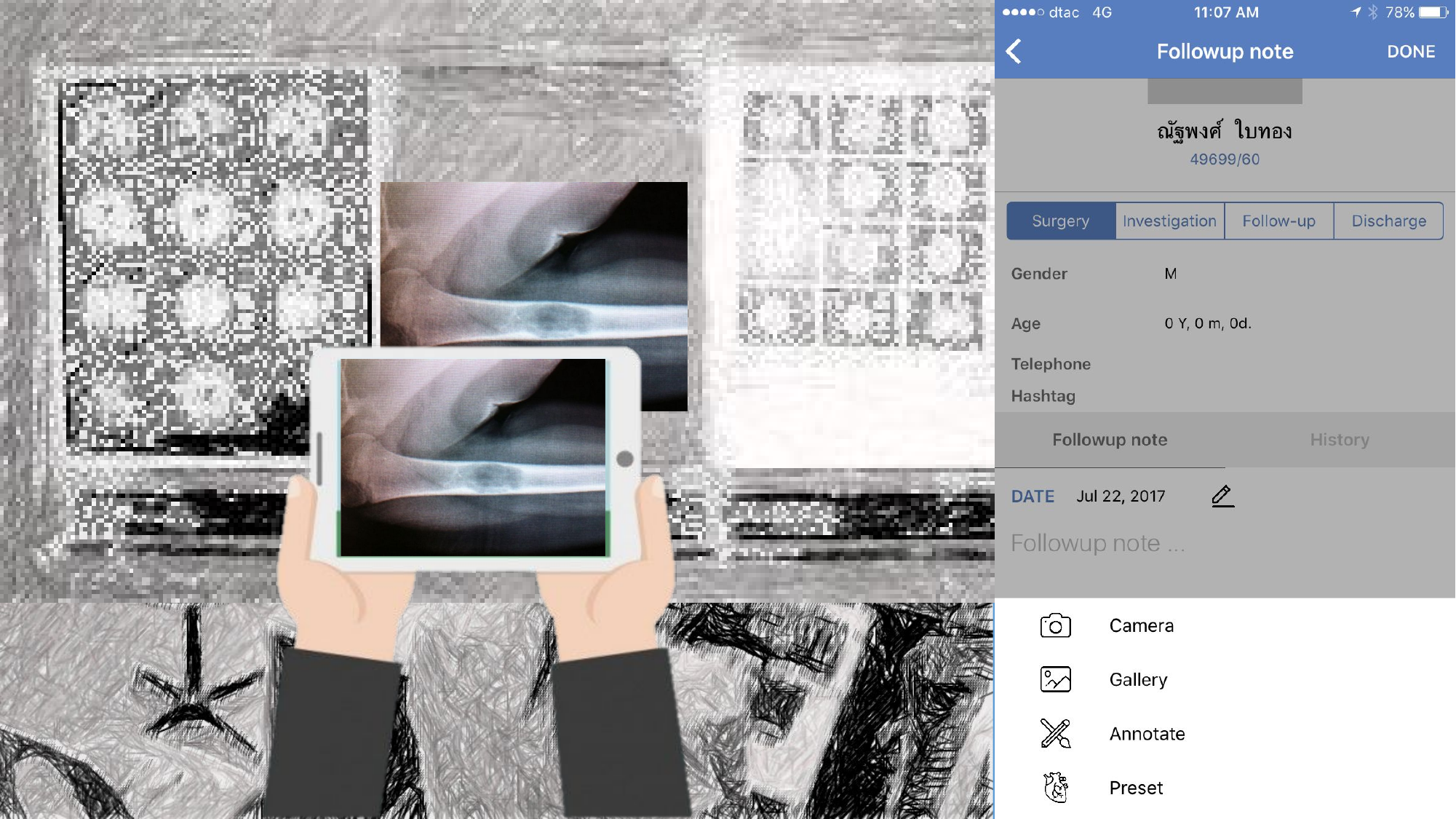

## Slide 5
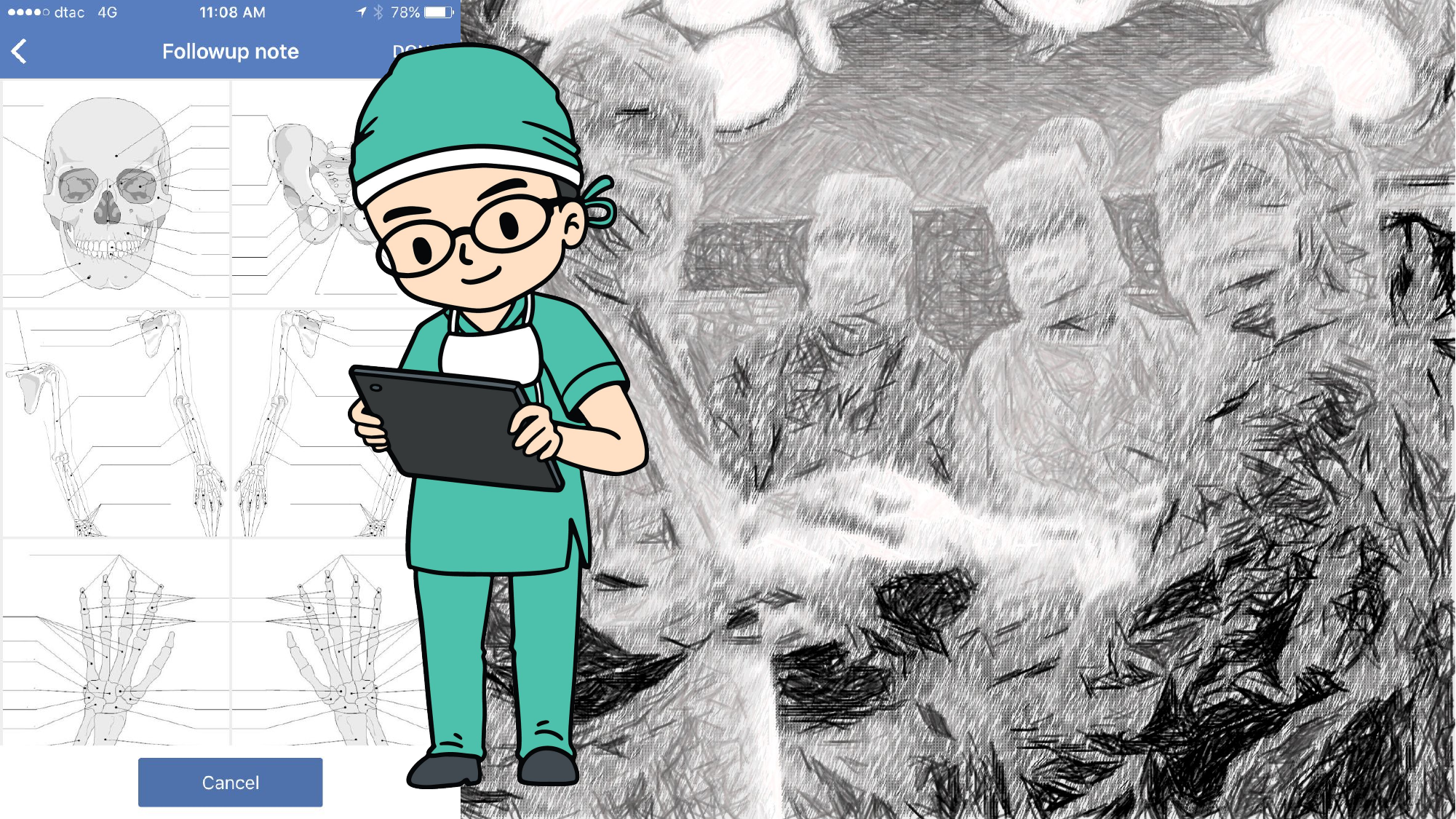

## Slide 6
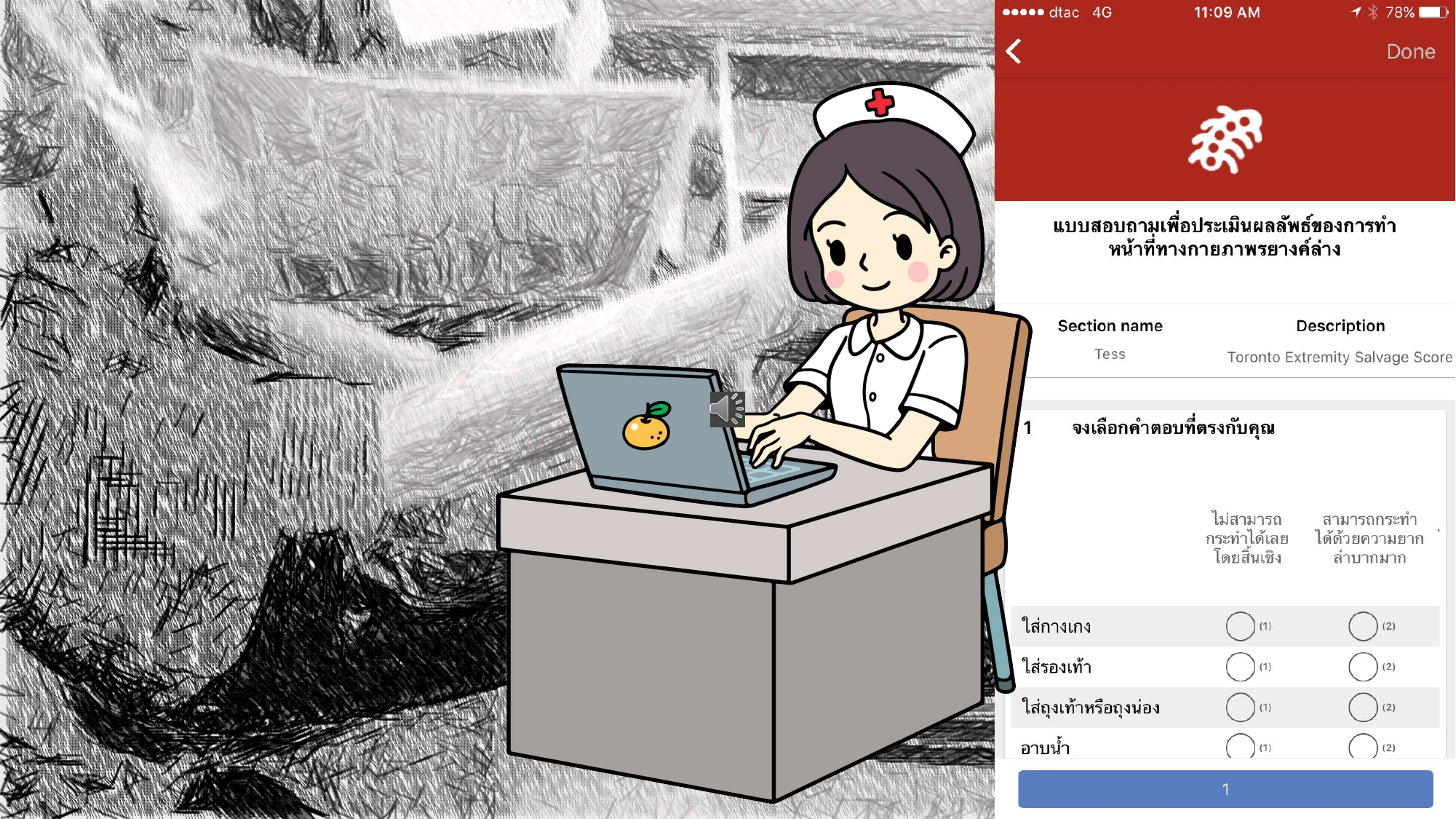

## Slide 7
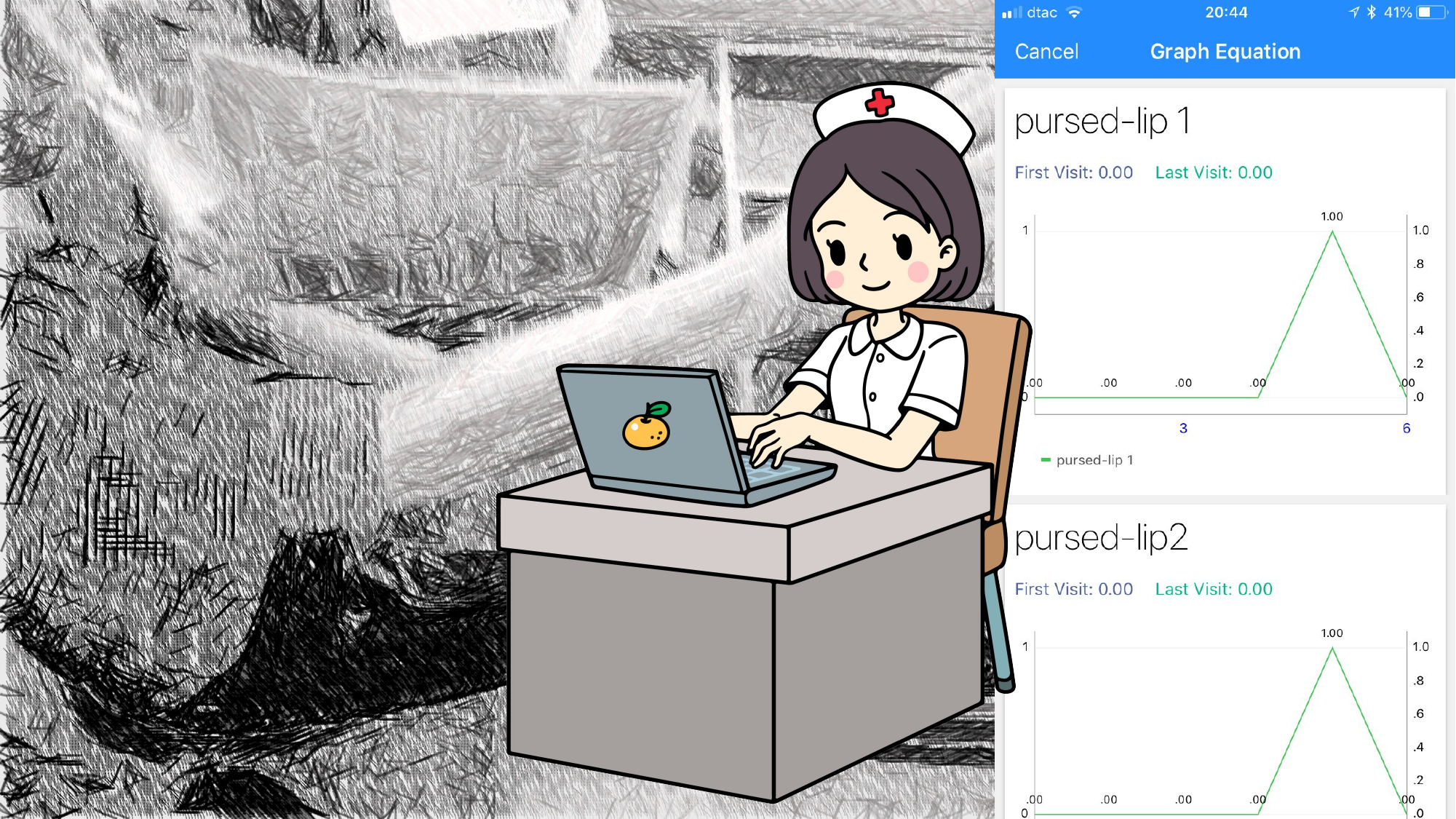

## Slide 8
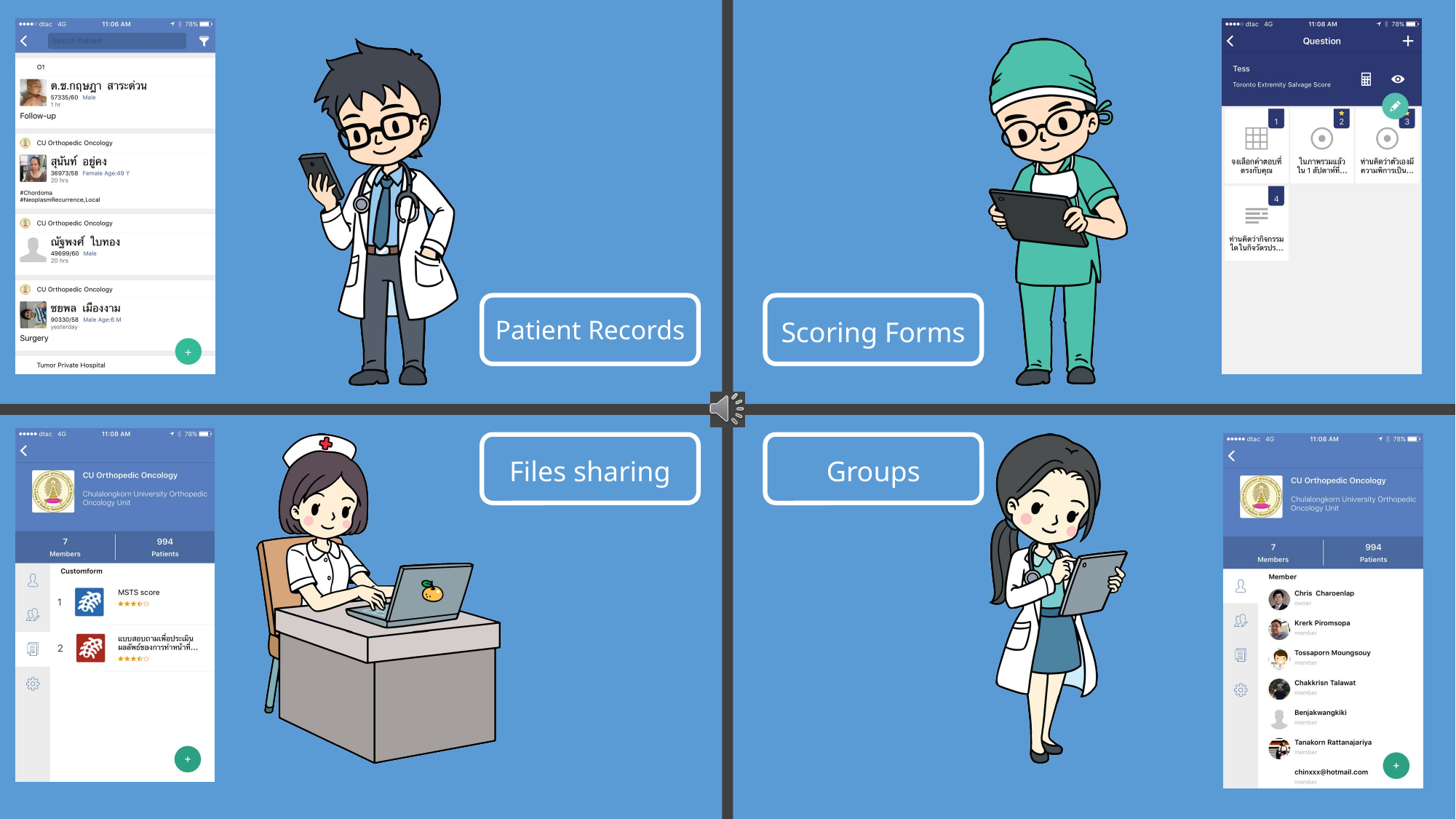

## Slide 9
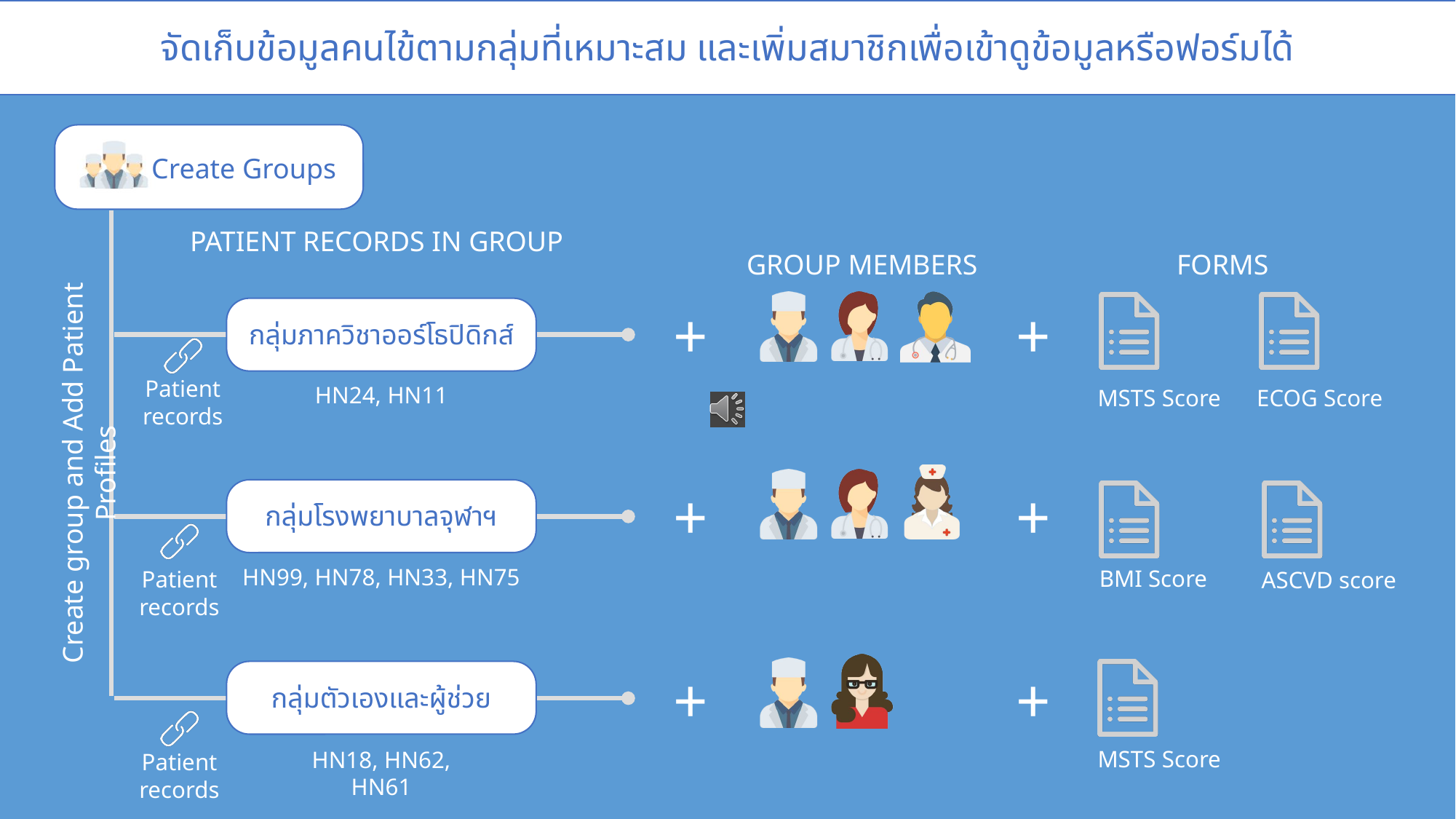

## Slide 10
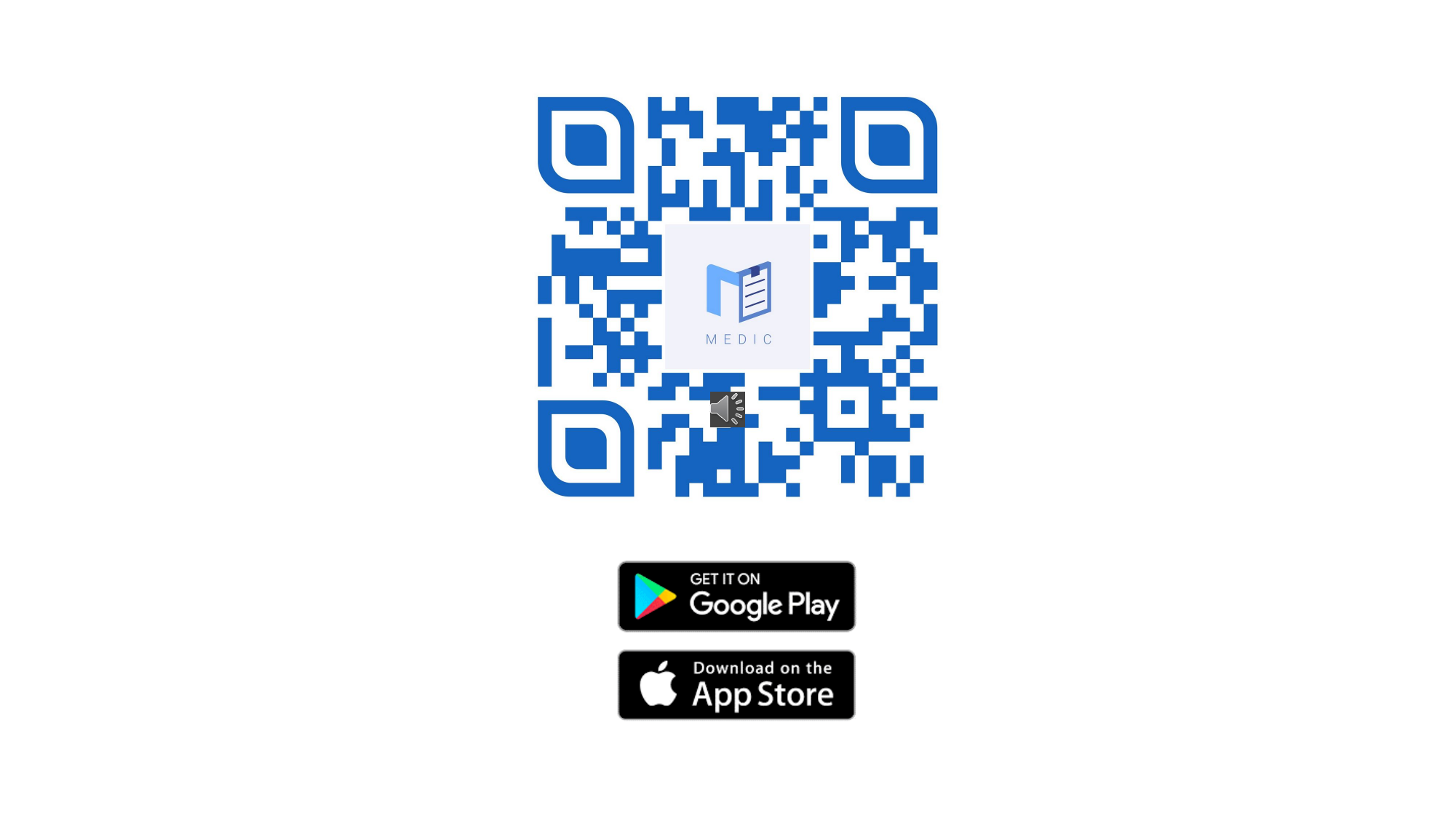

## Slide 11
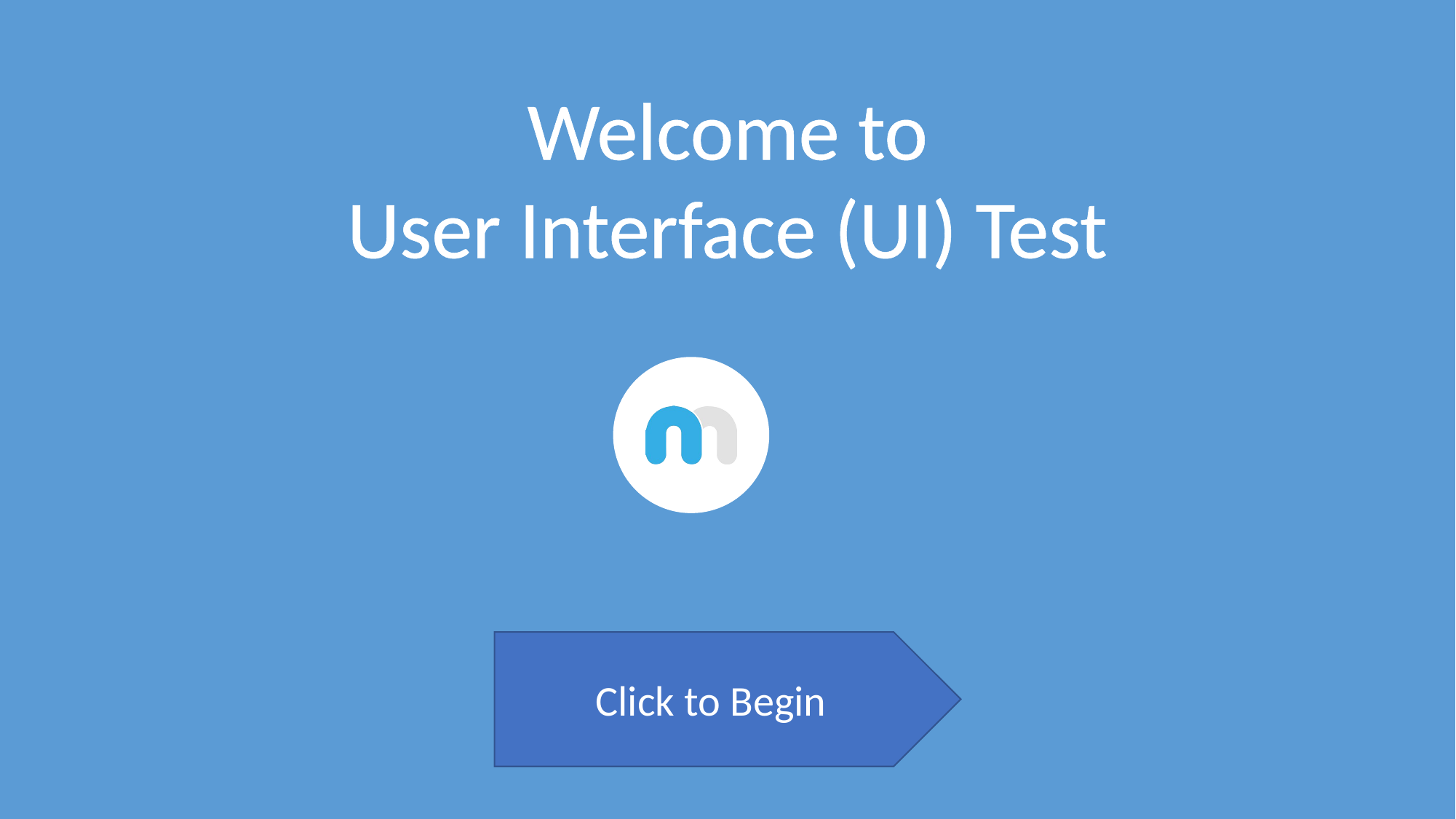

## Slide 12
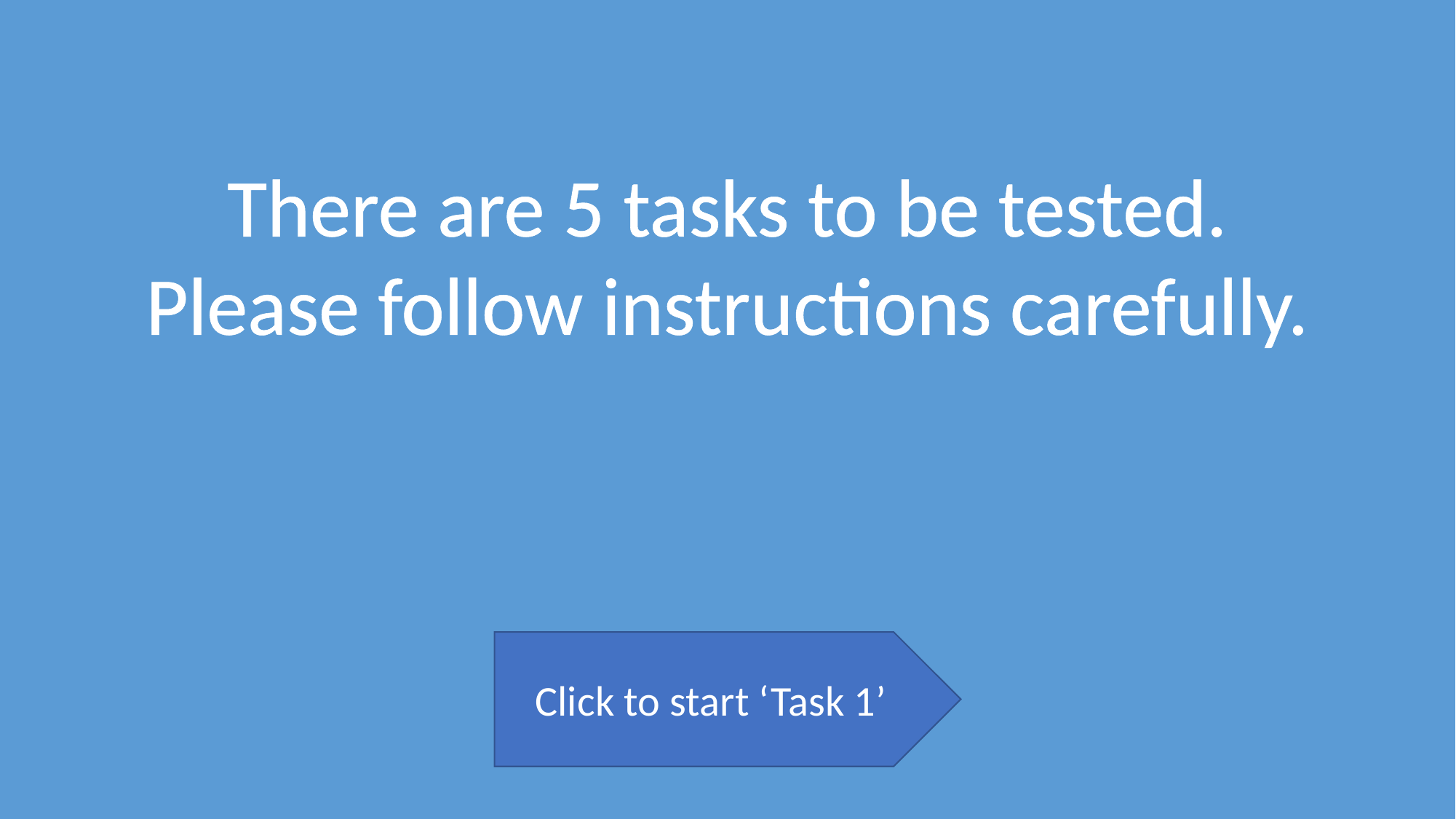

## Slide 13
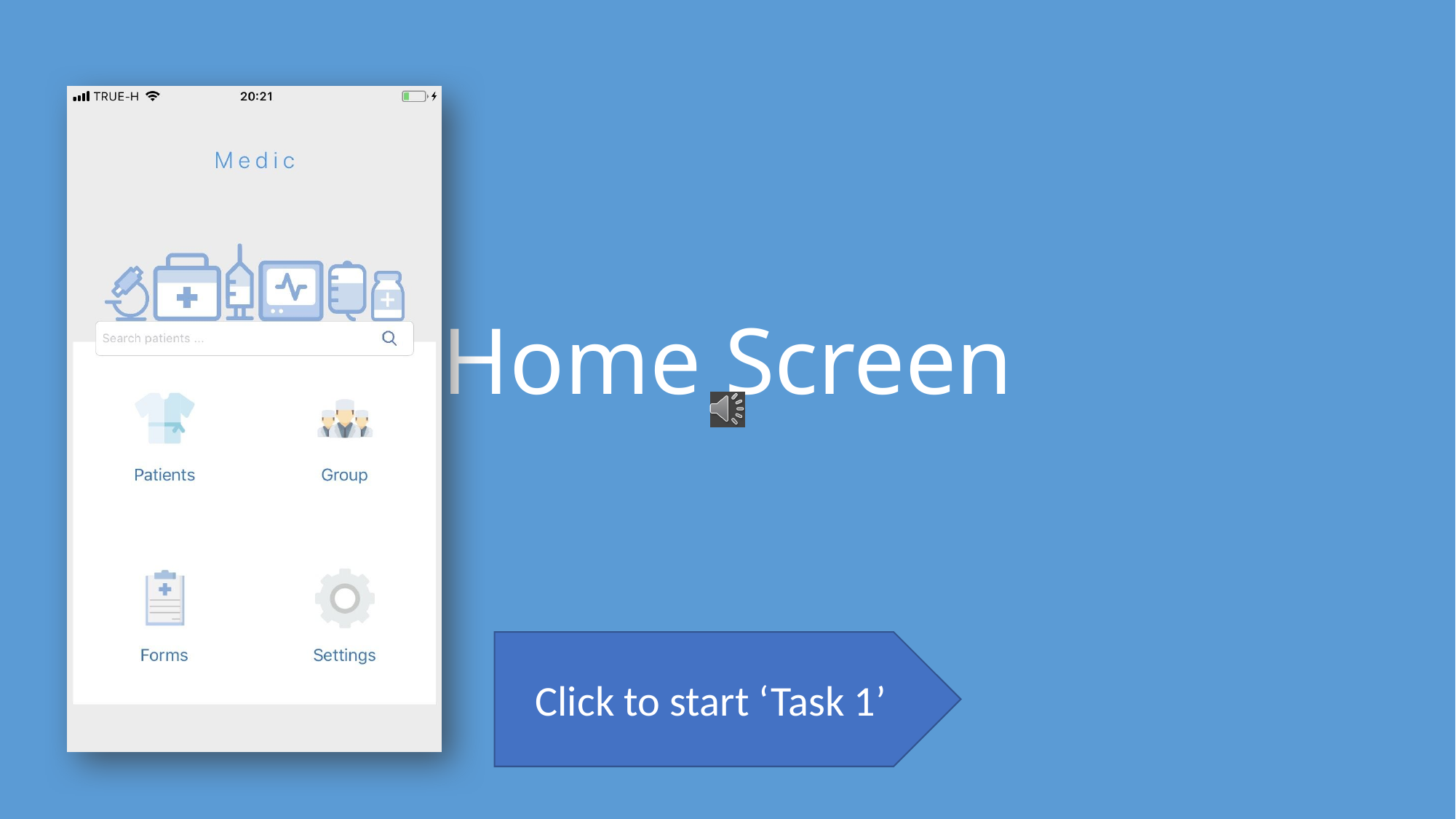

## Slide 14
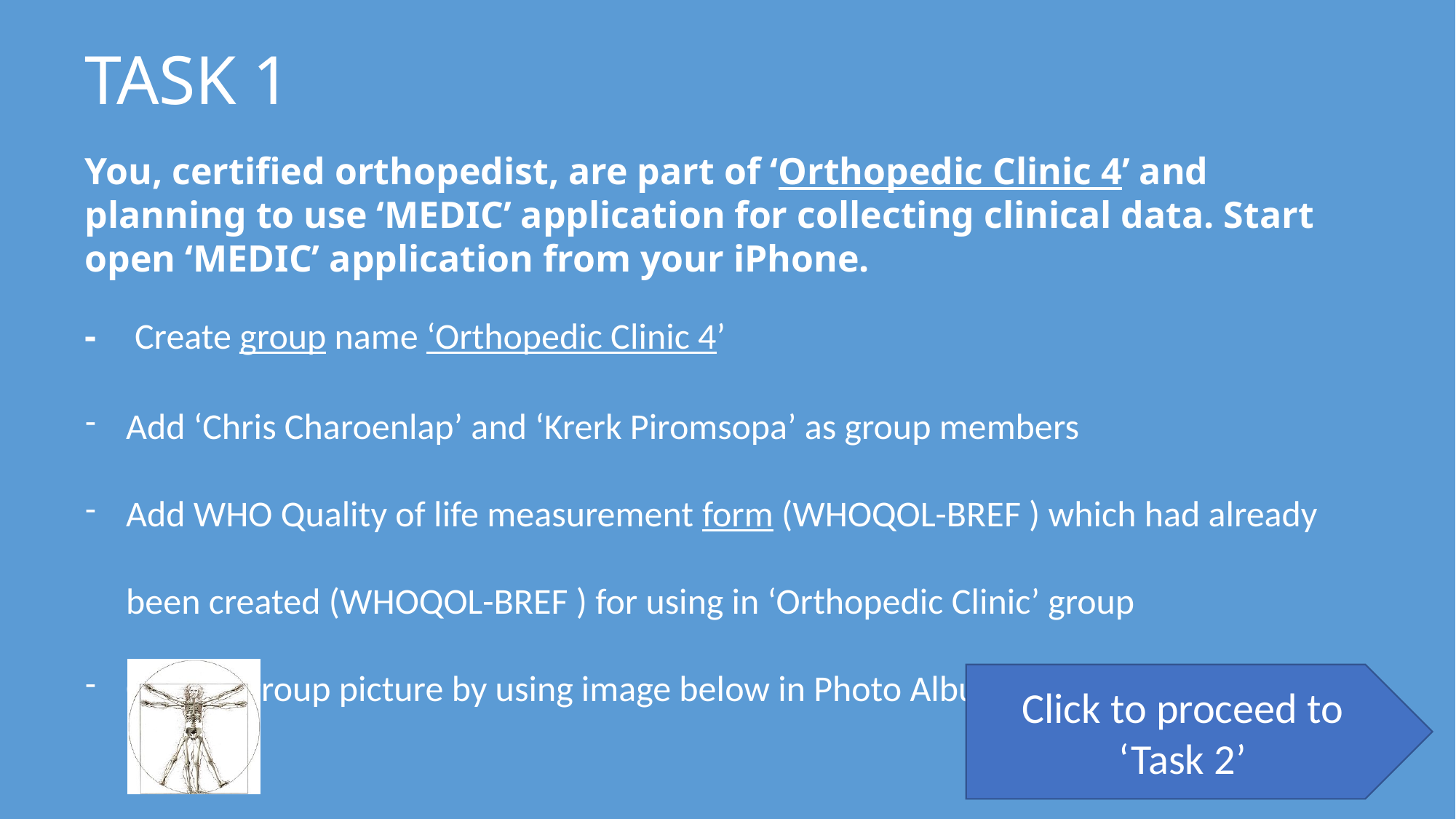

## Slide 15
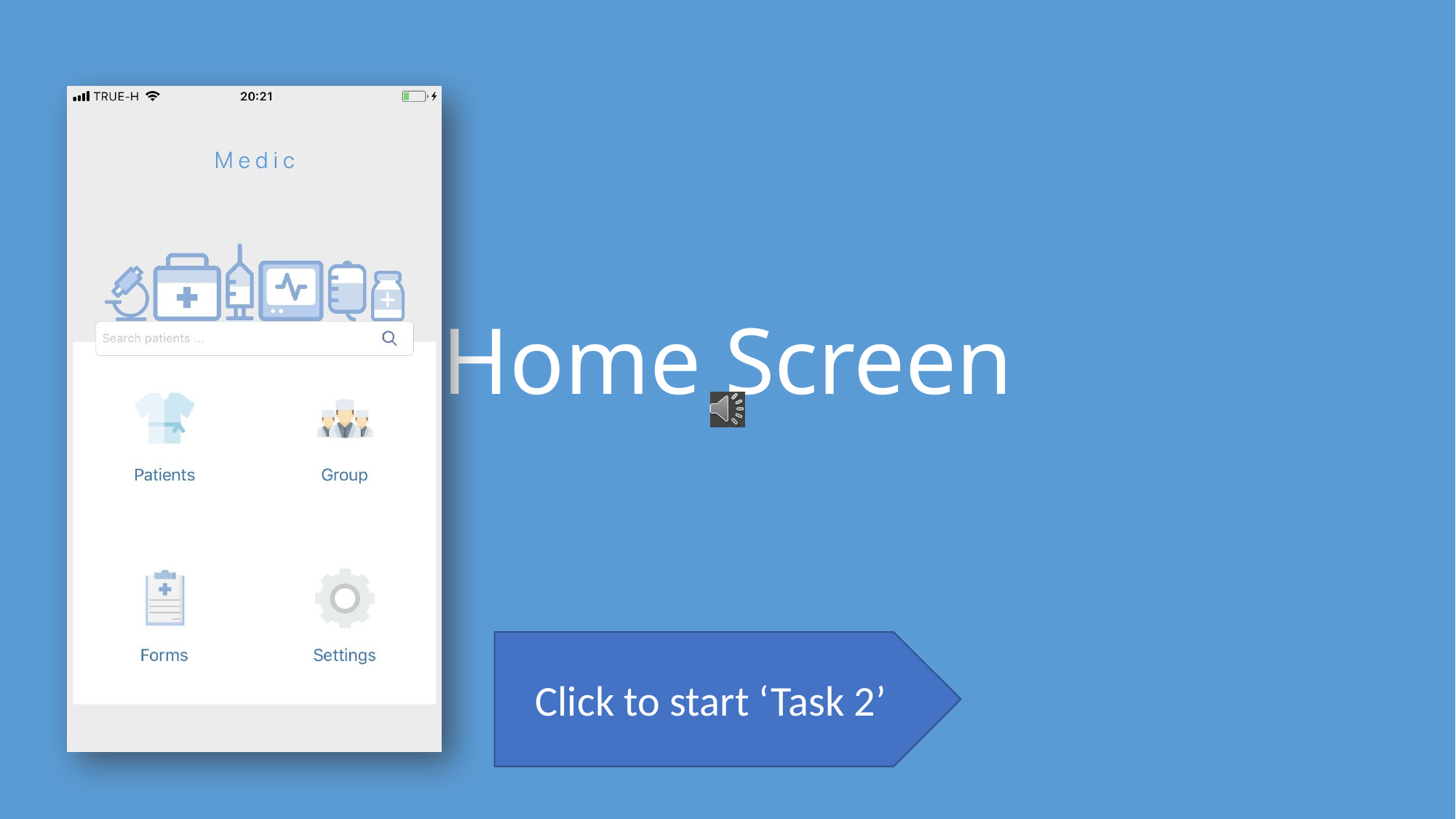

## Slide 16
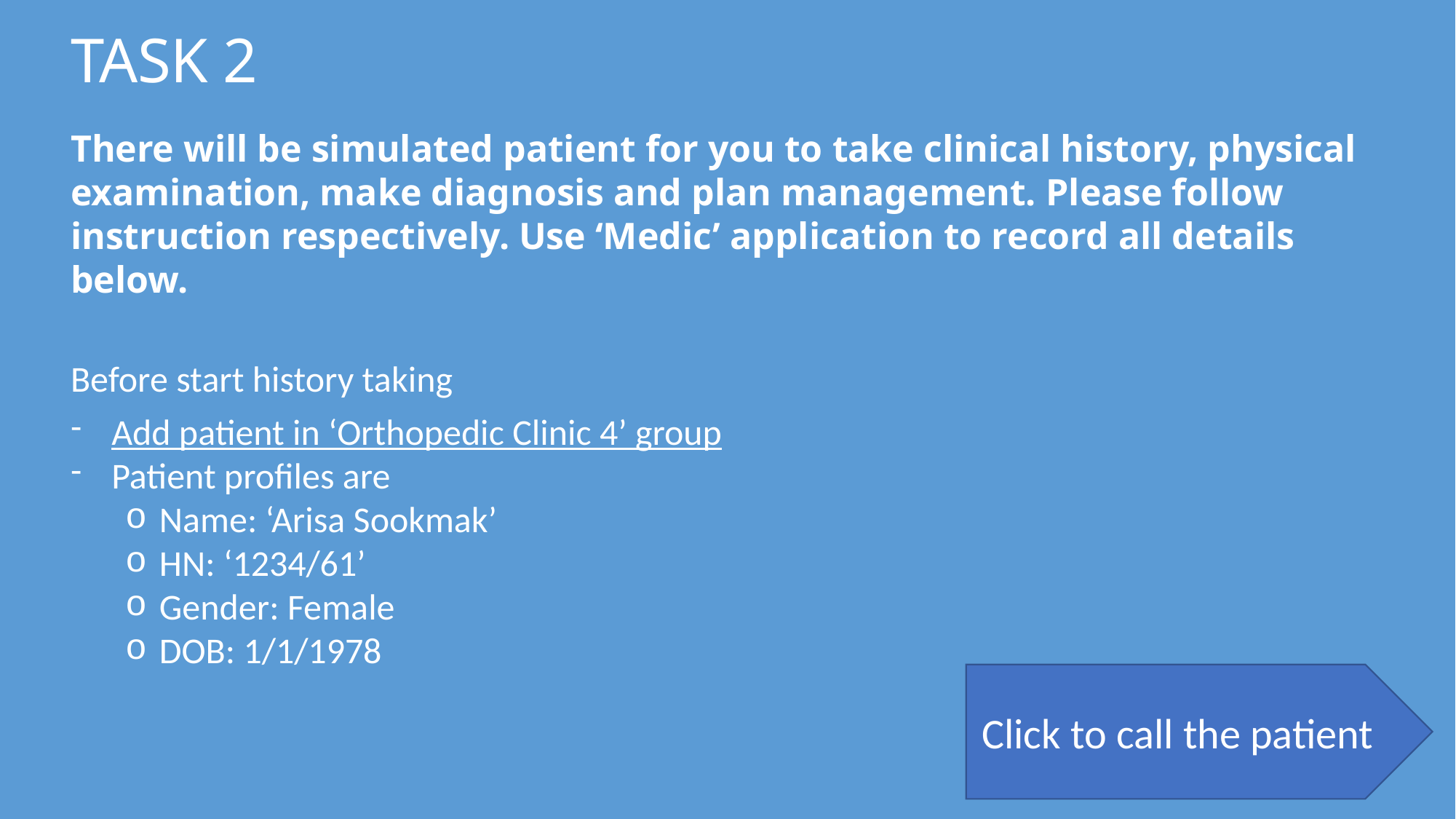

## Slide 17
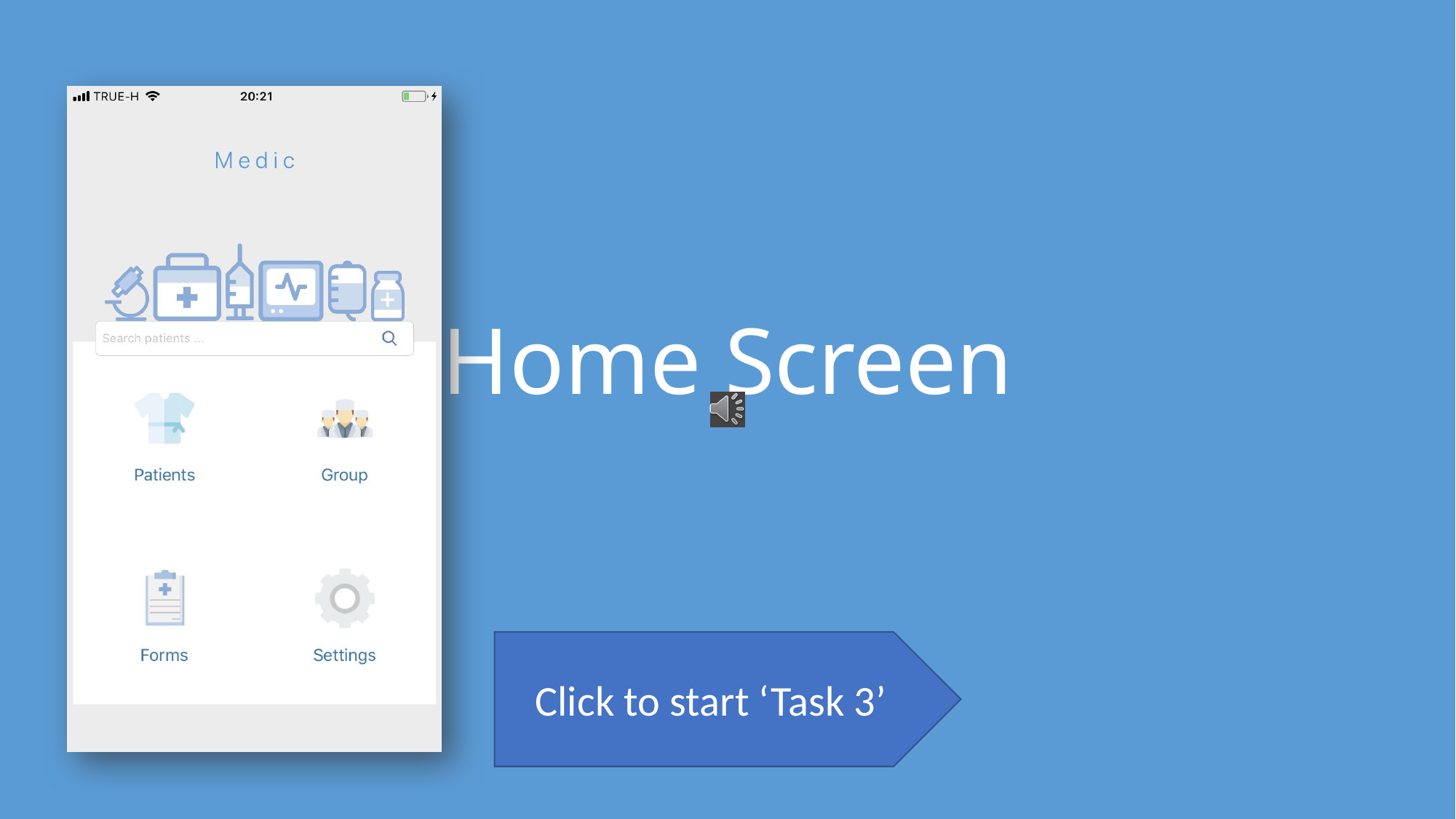

## Slide 18
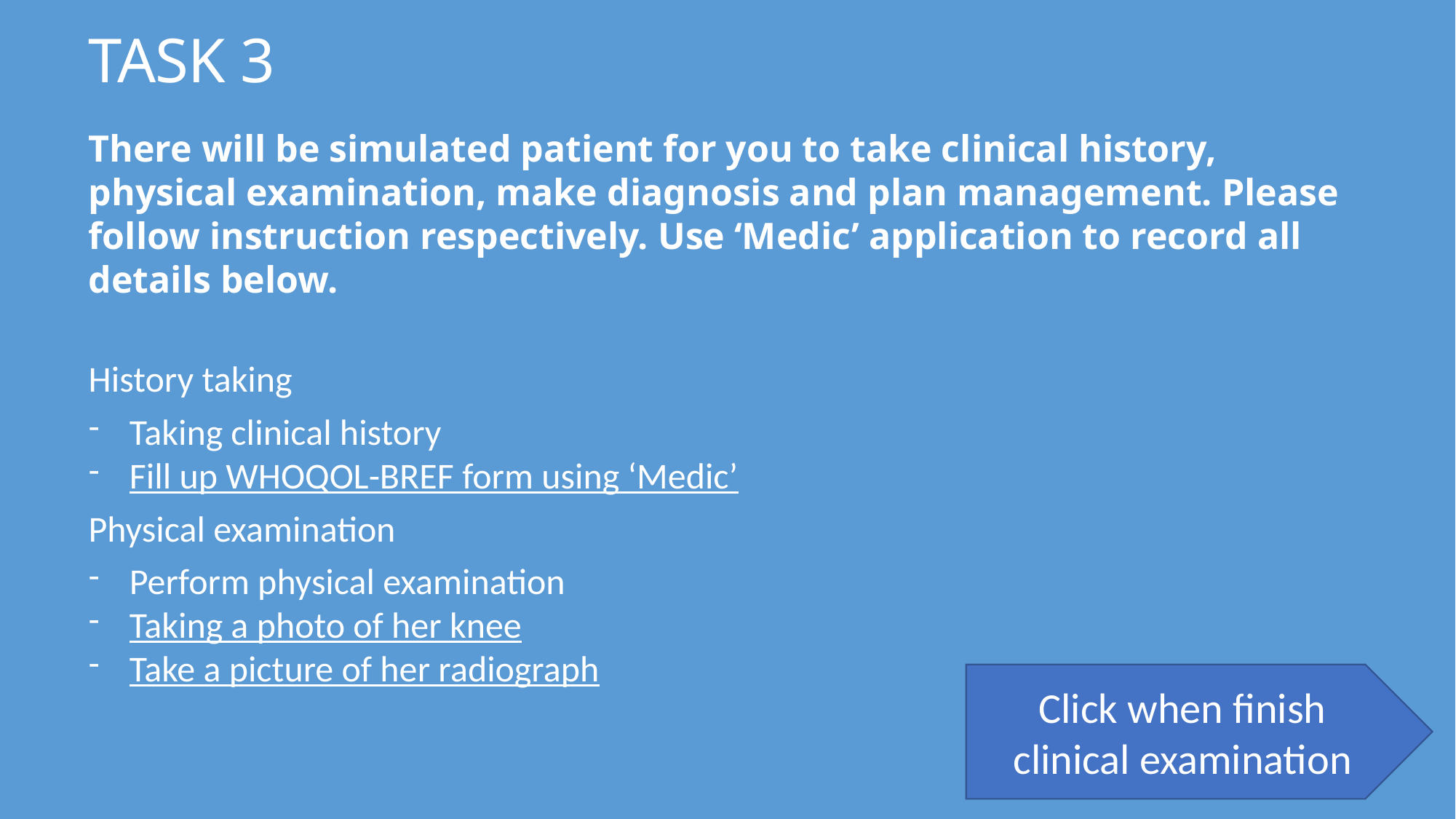

## Slide 19
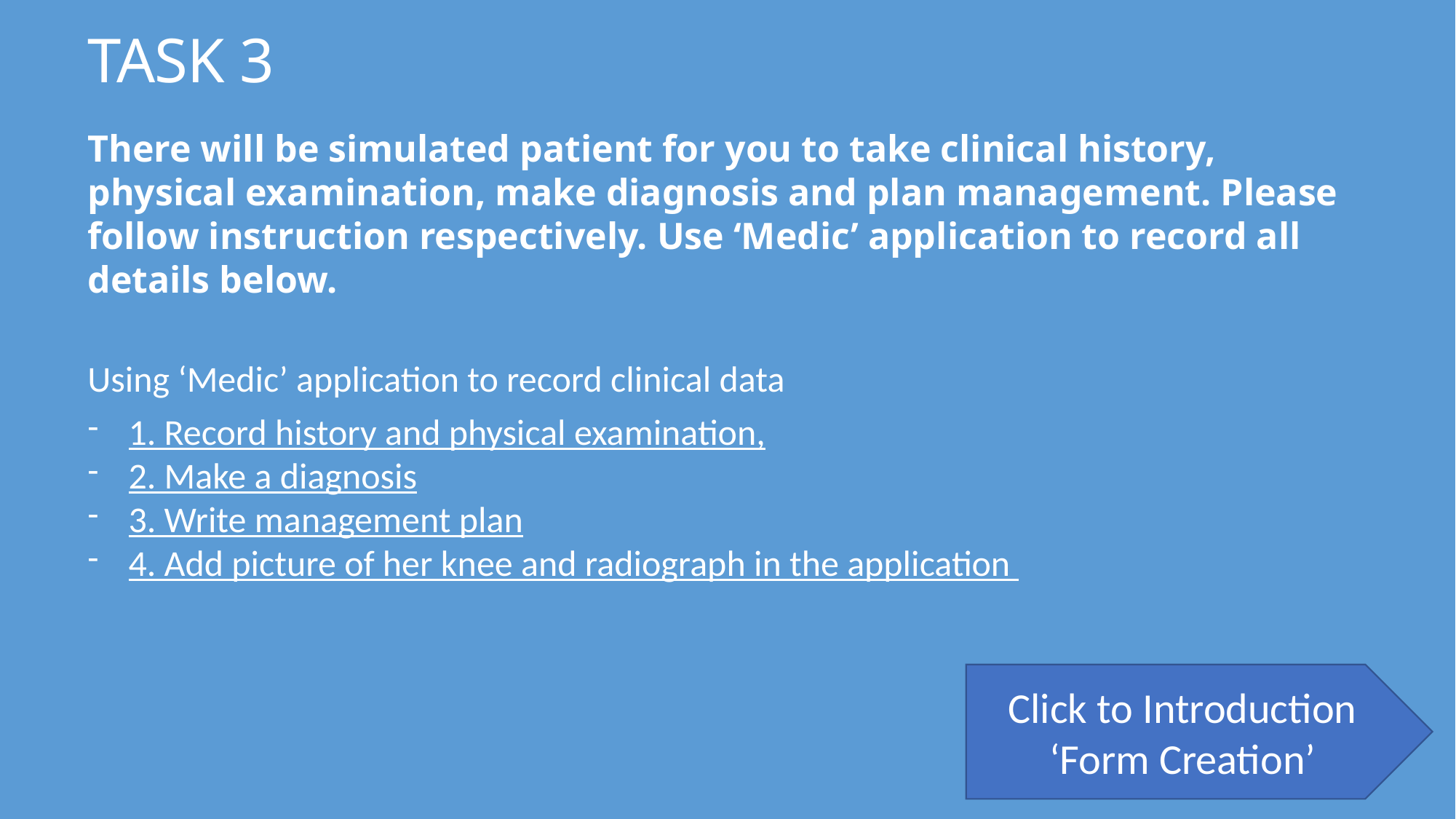

## Slide 20
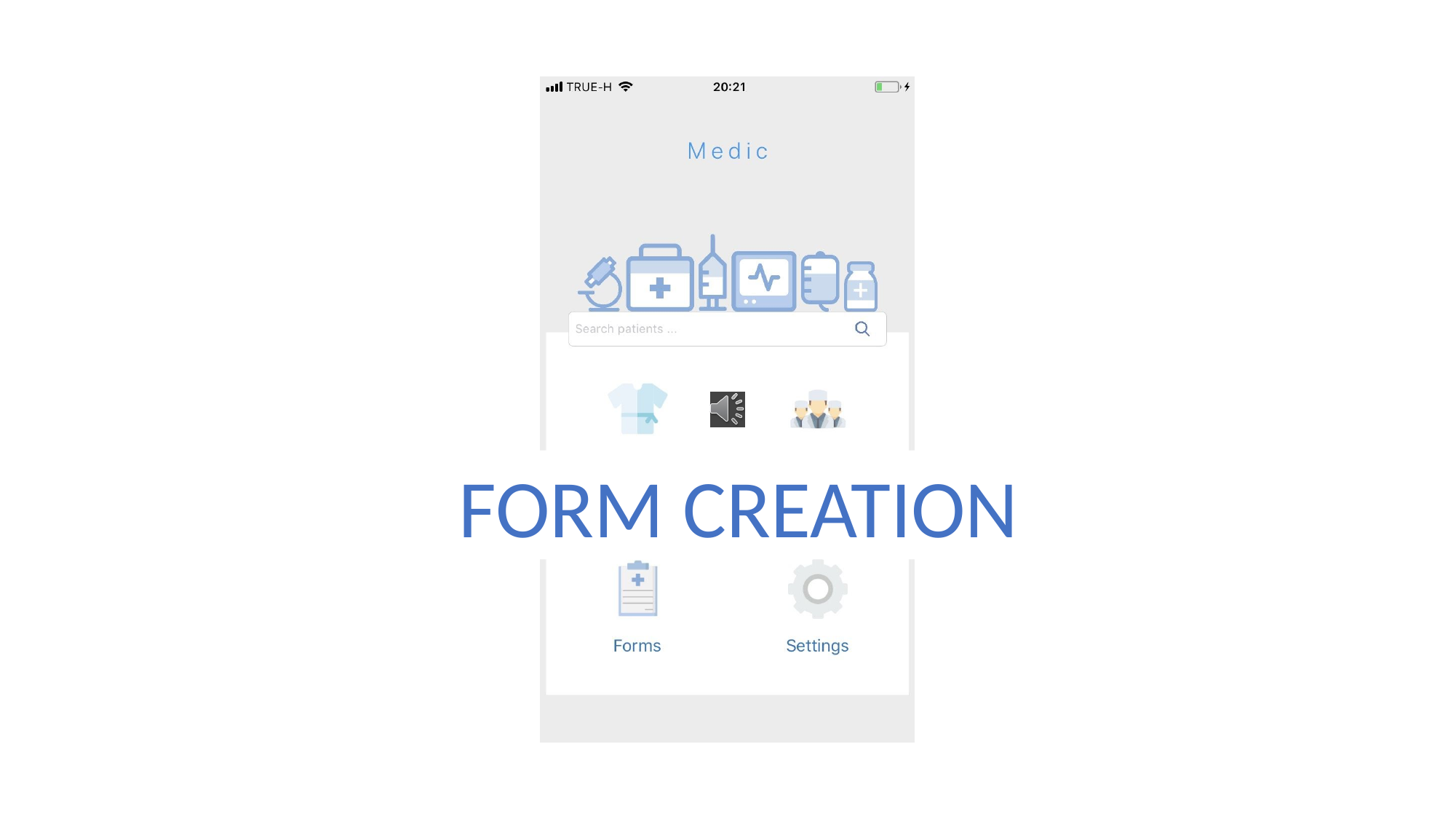

## Slide 21
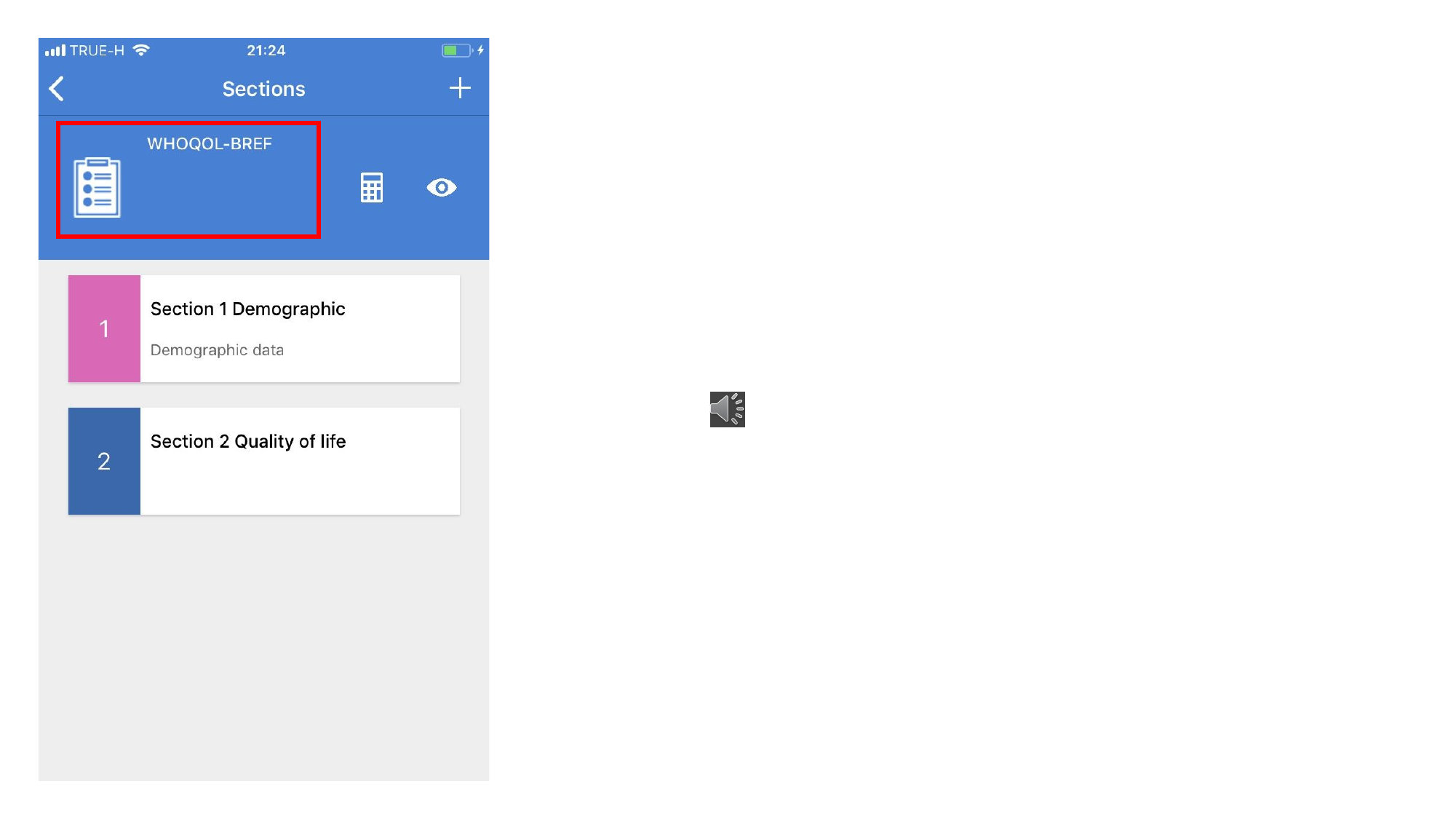

## Slide 22
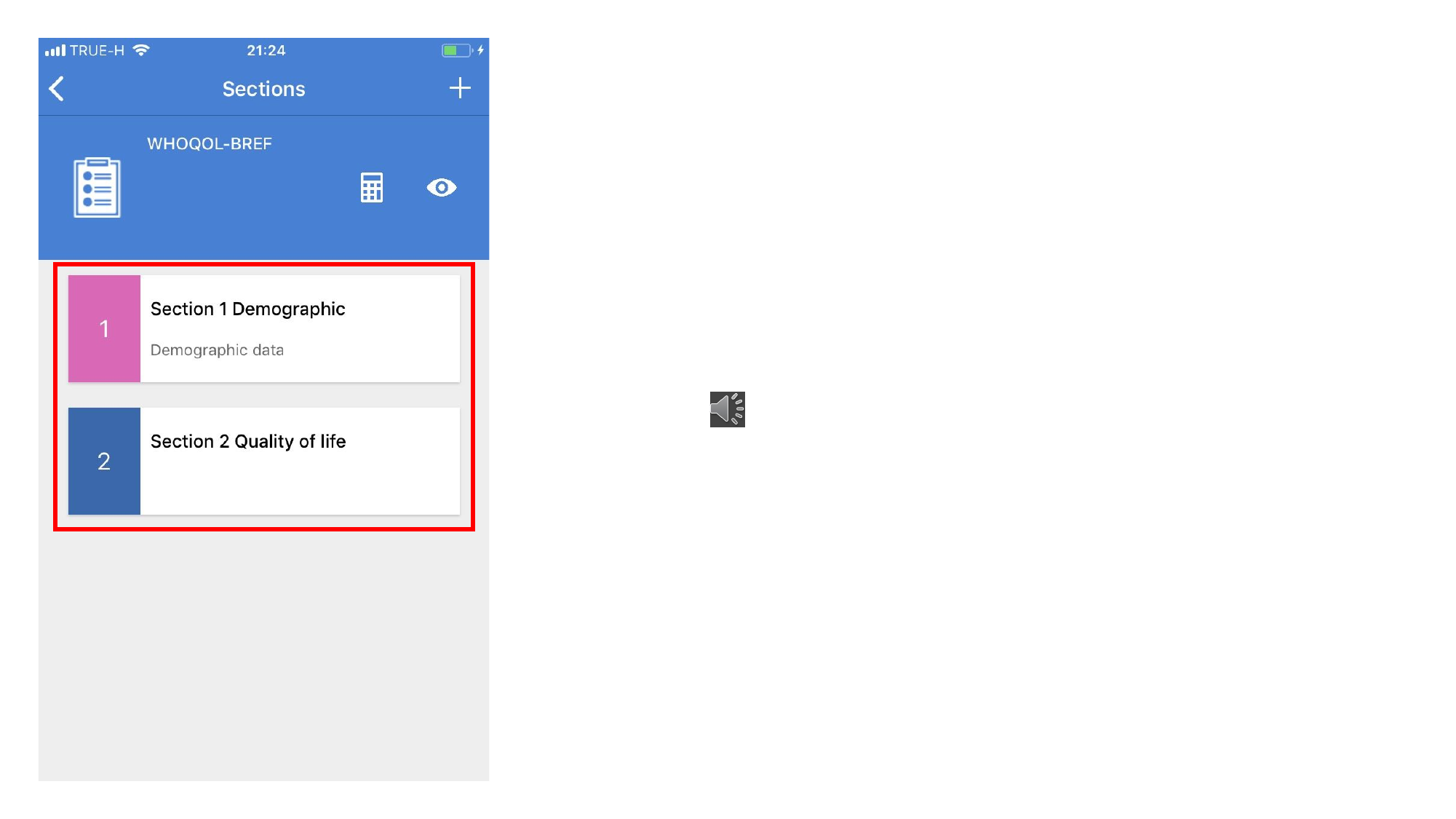

## Slide 23
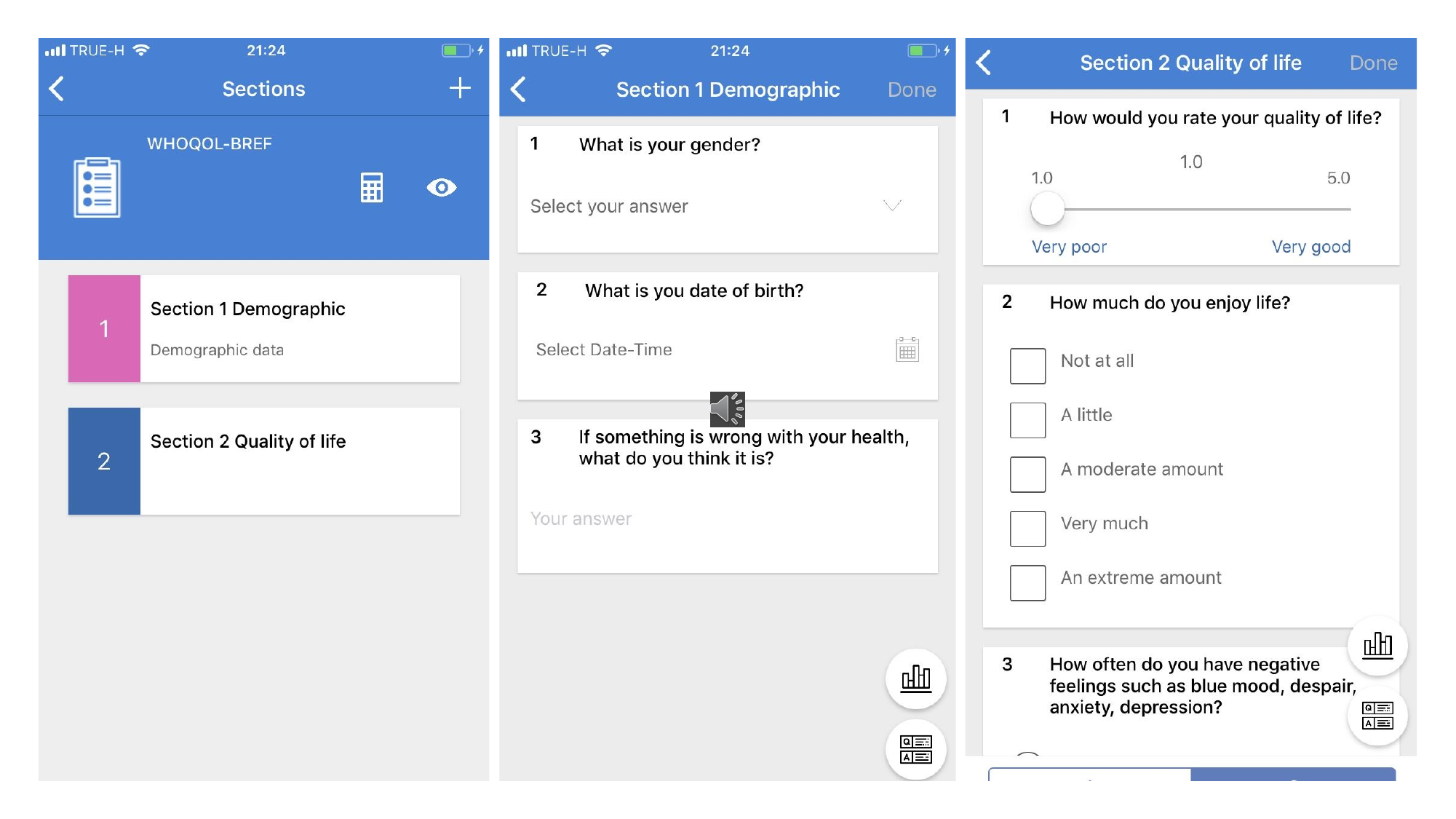

## Slide 24
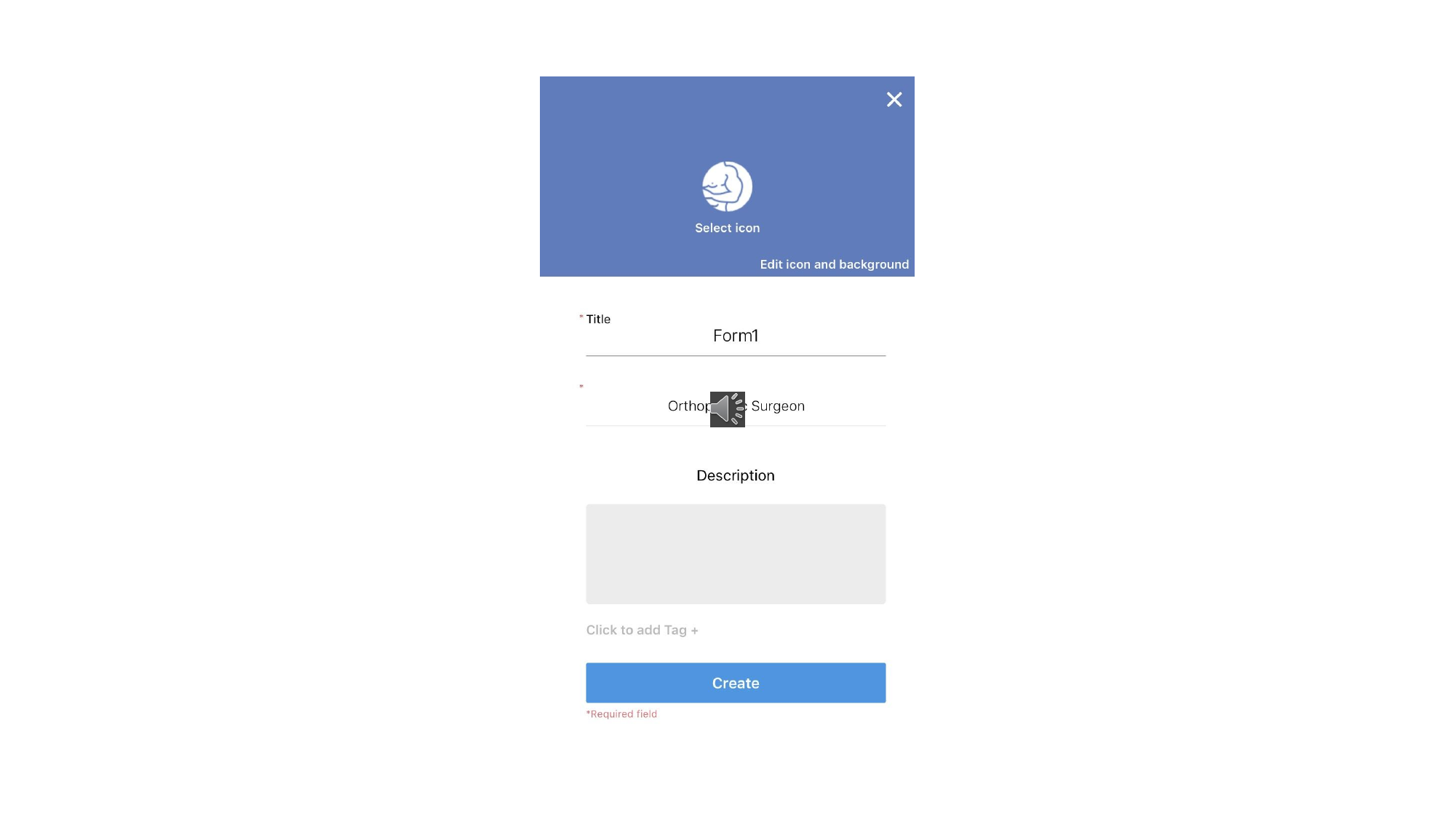

## Slide 25
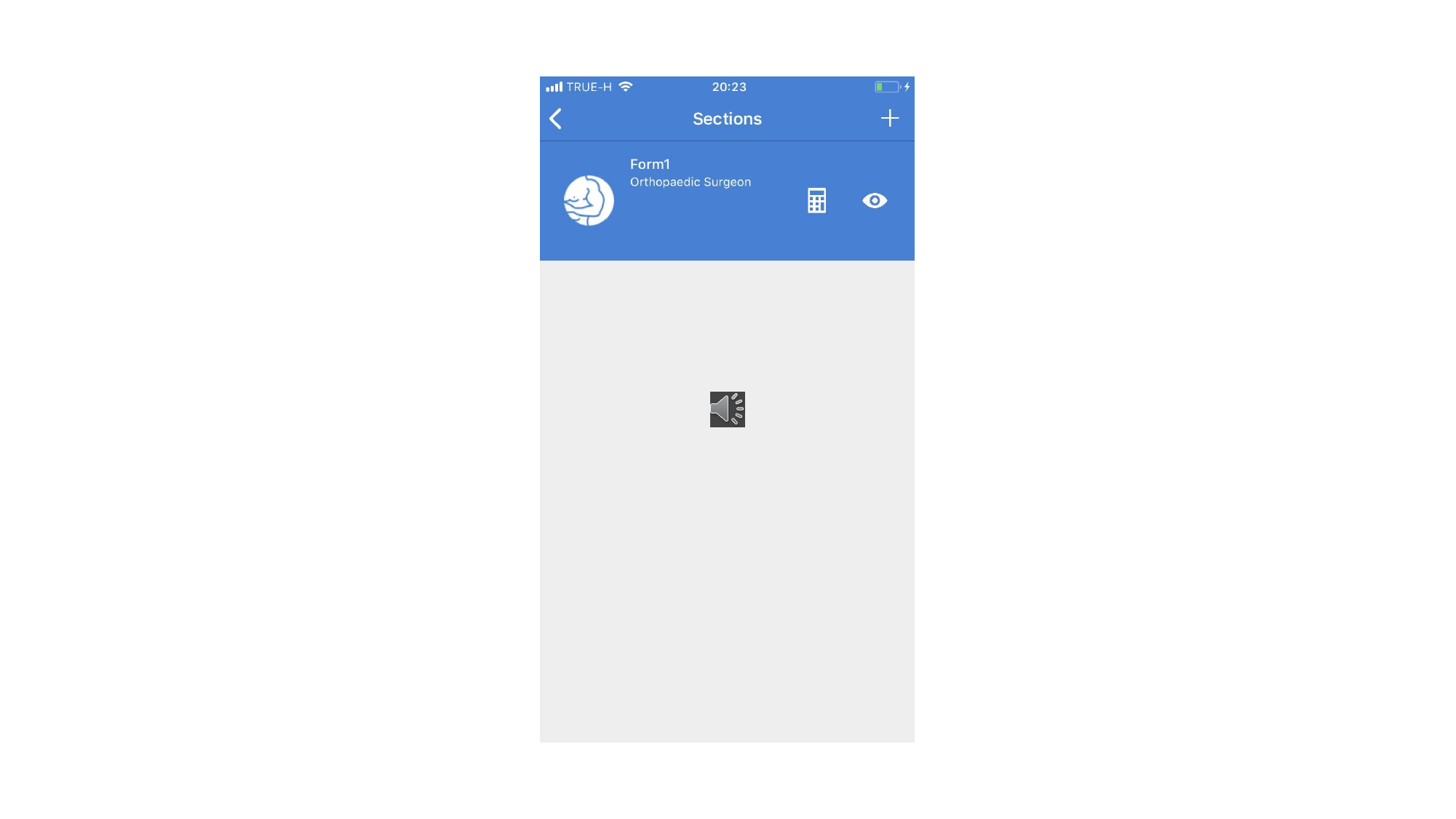

## Slide 26
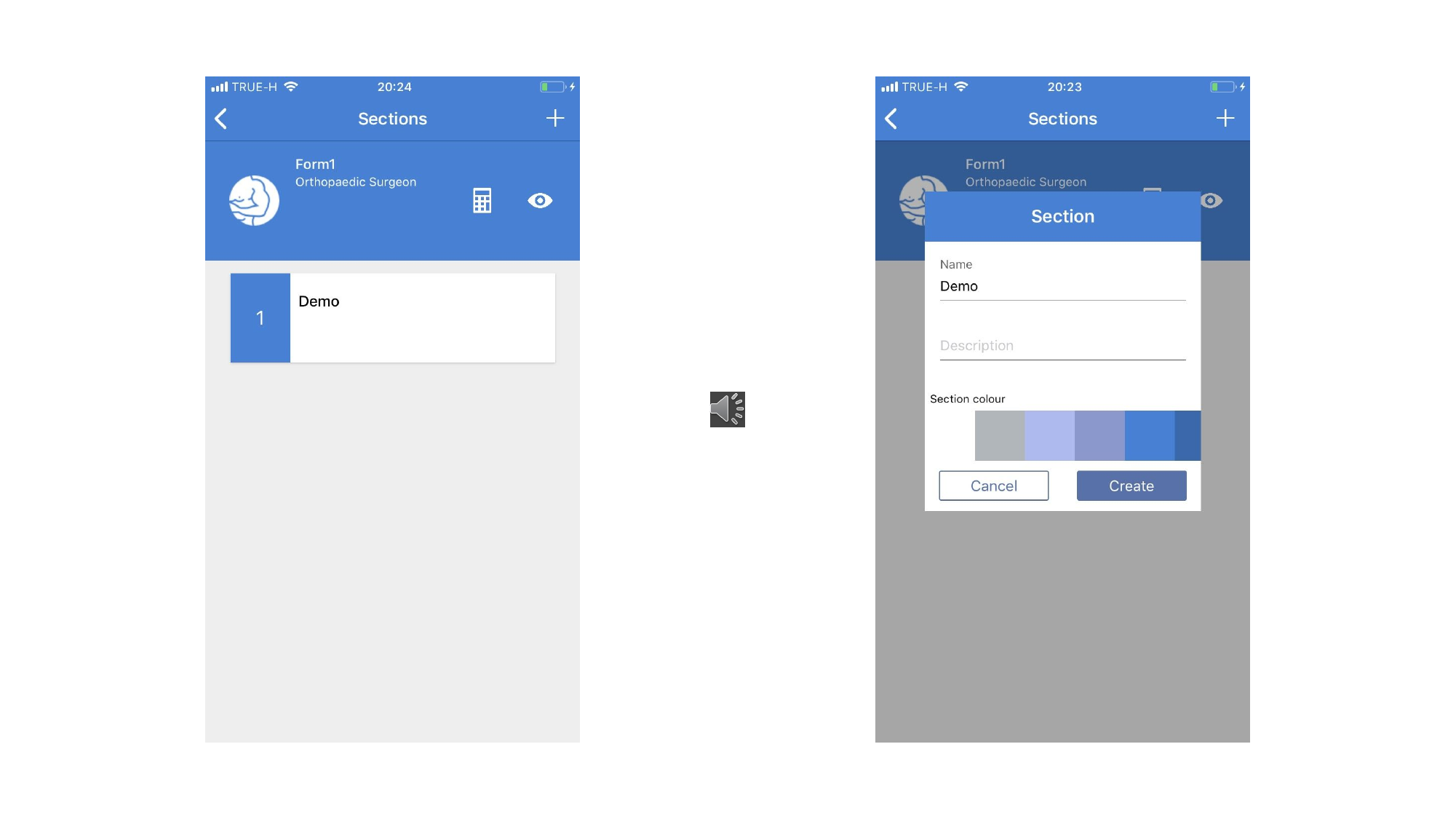

## Slide 27
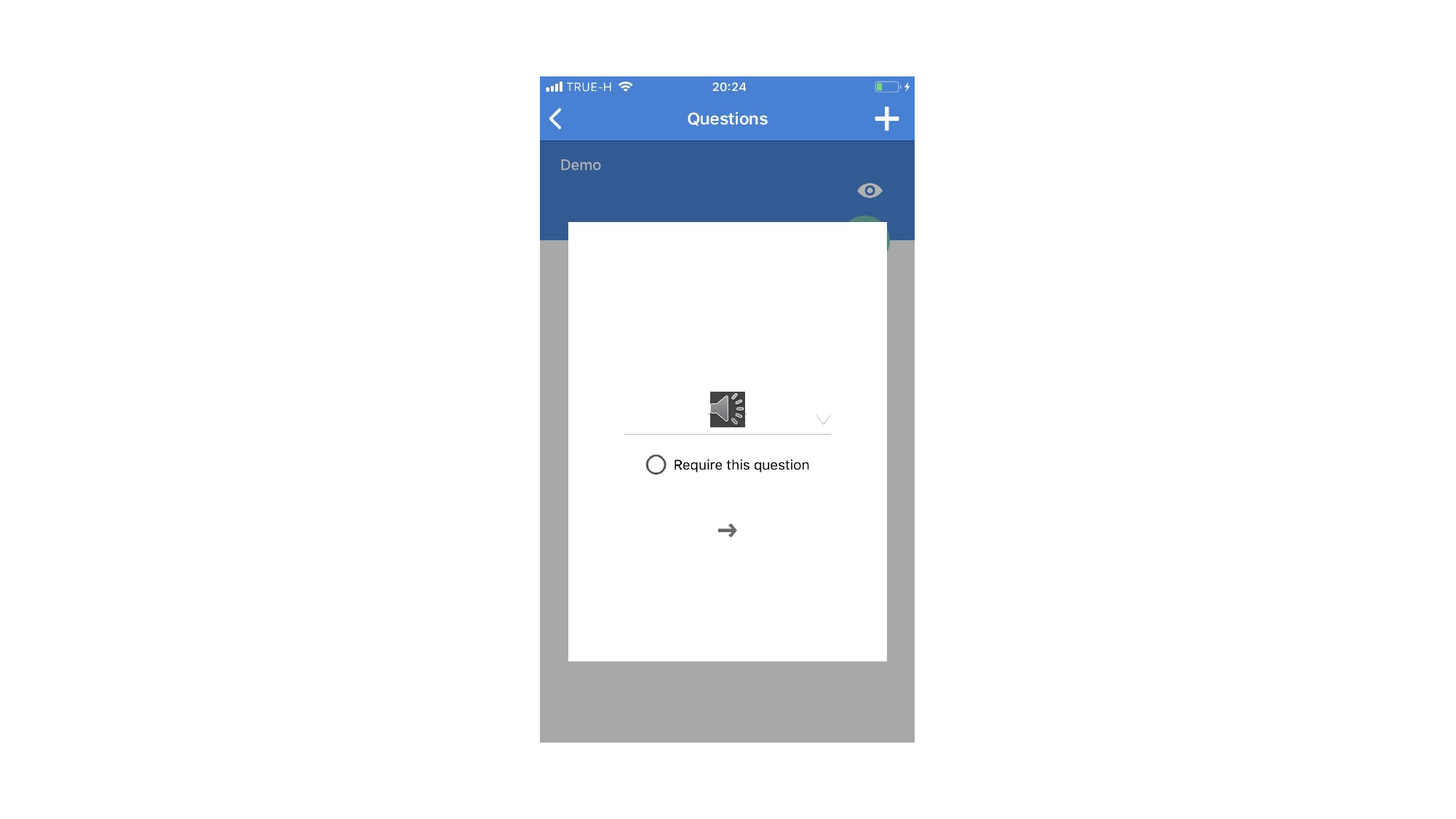

## Slide 28
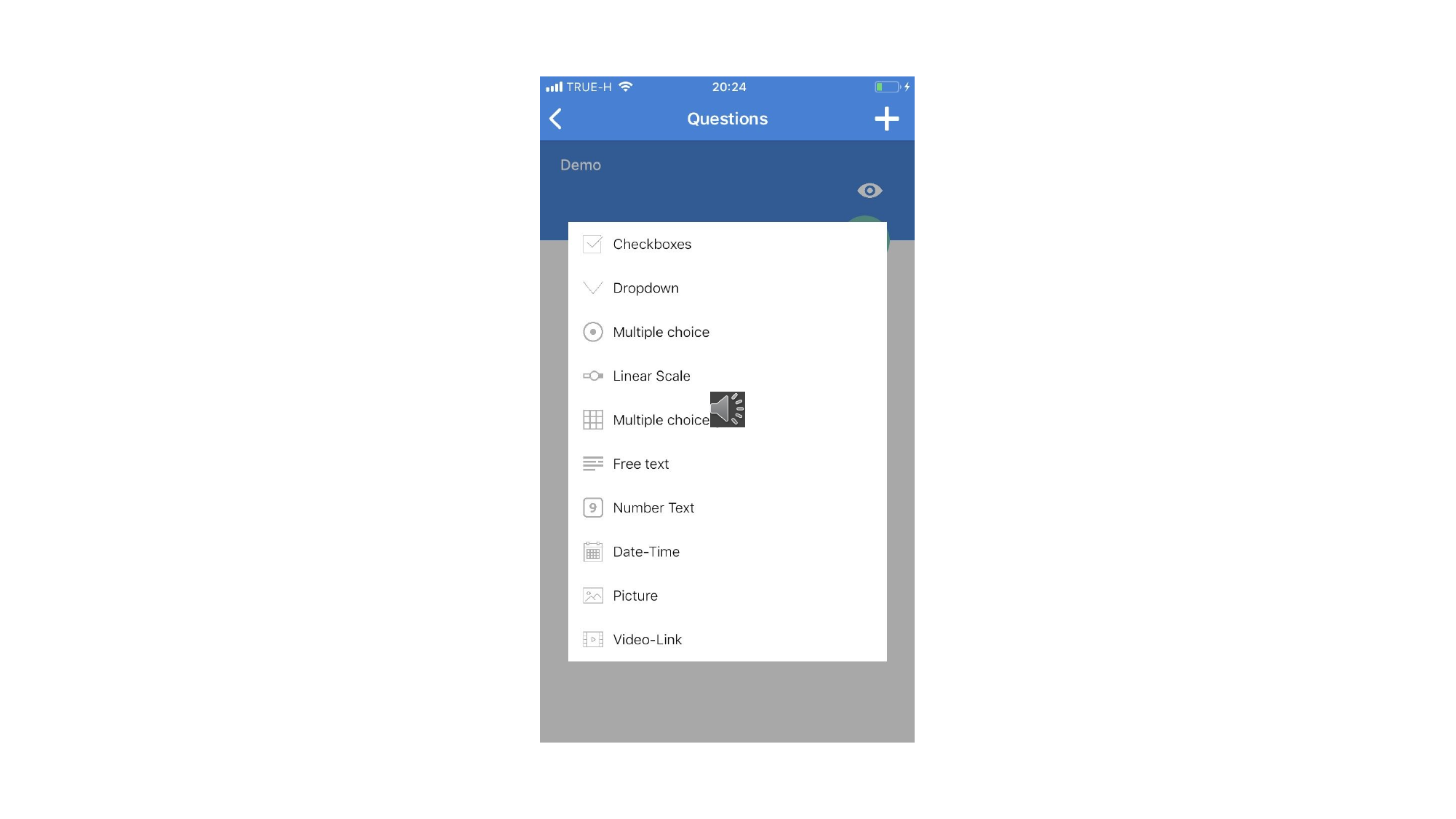

## Slide 29
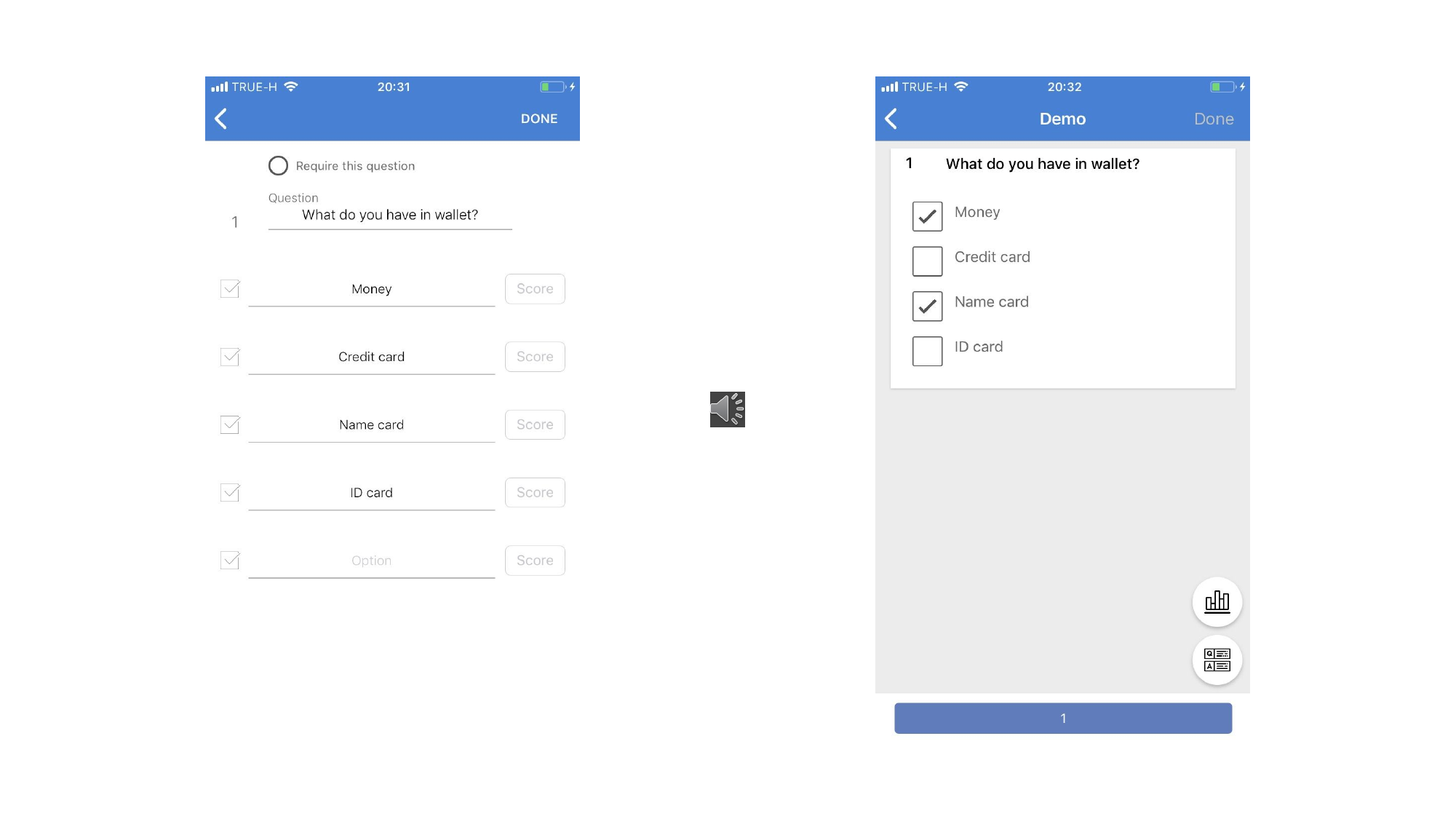

## Slide 30
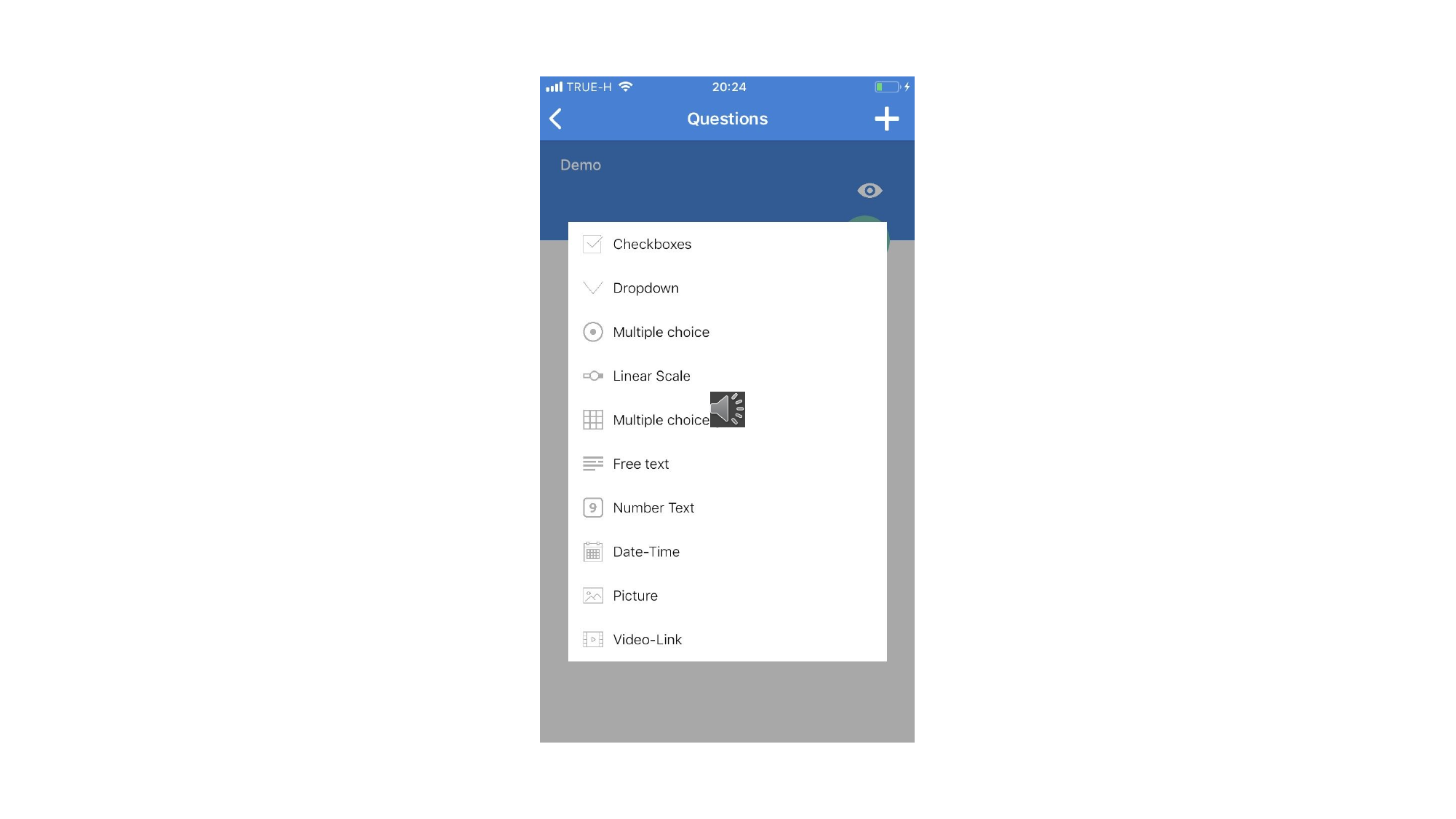

## Slide 31
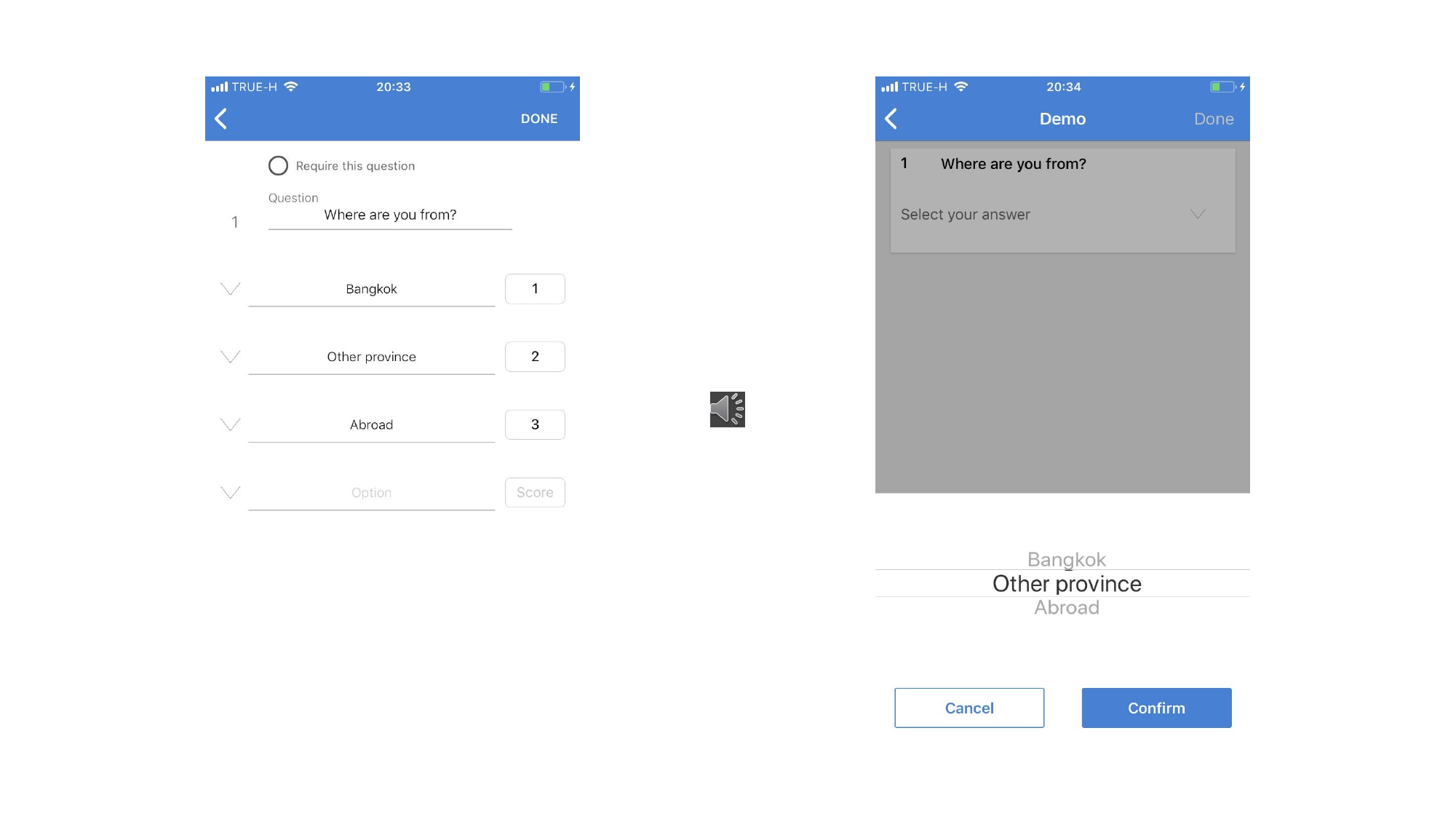

## Slide 32
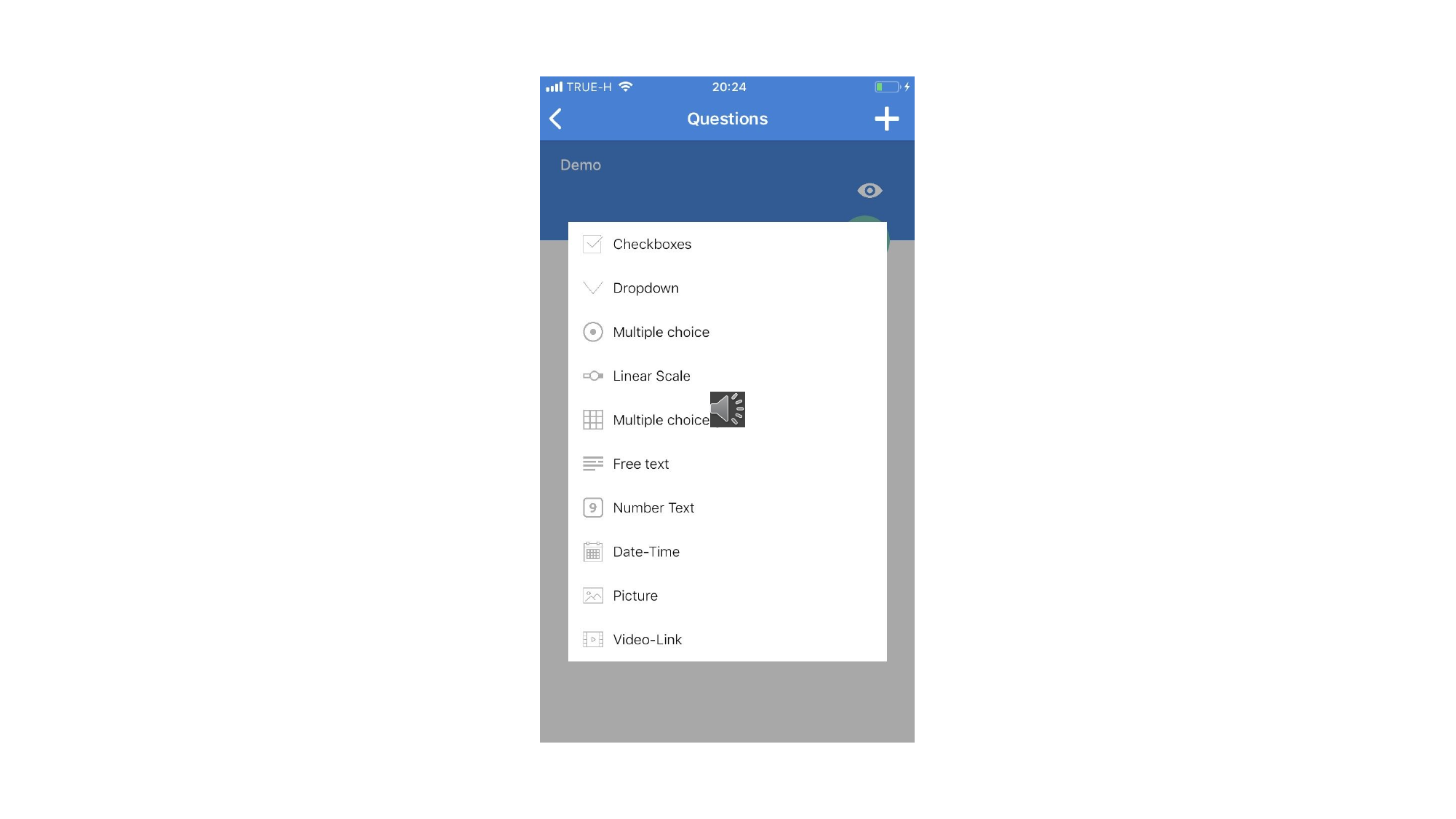

## Slide 33
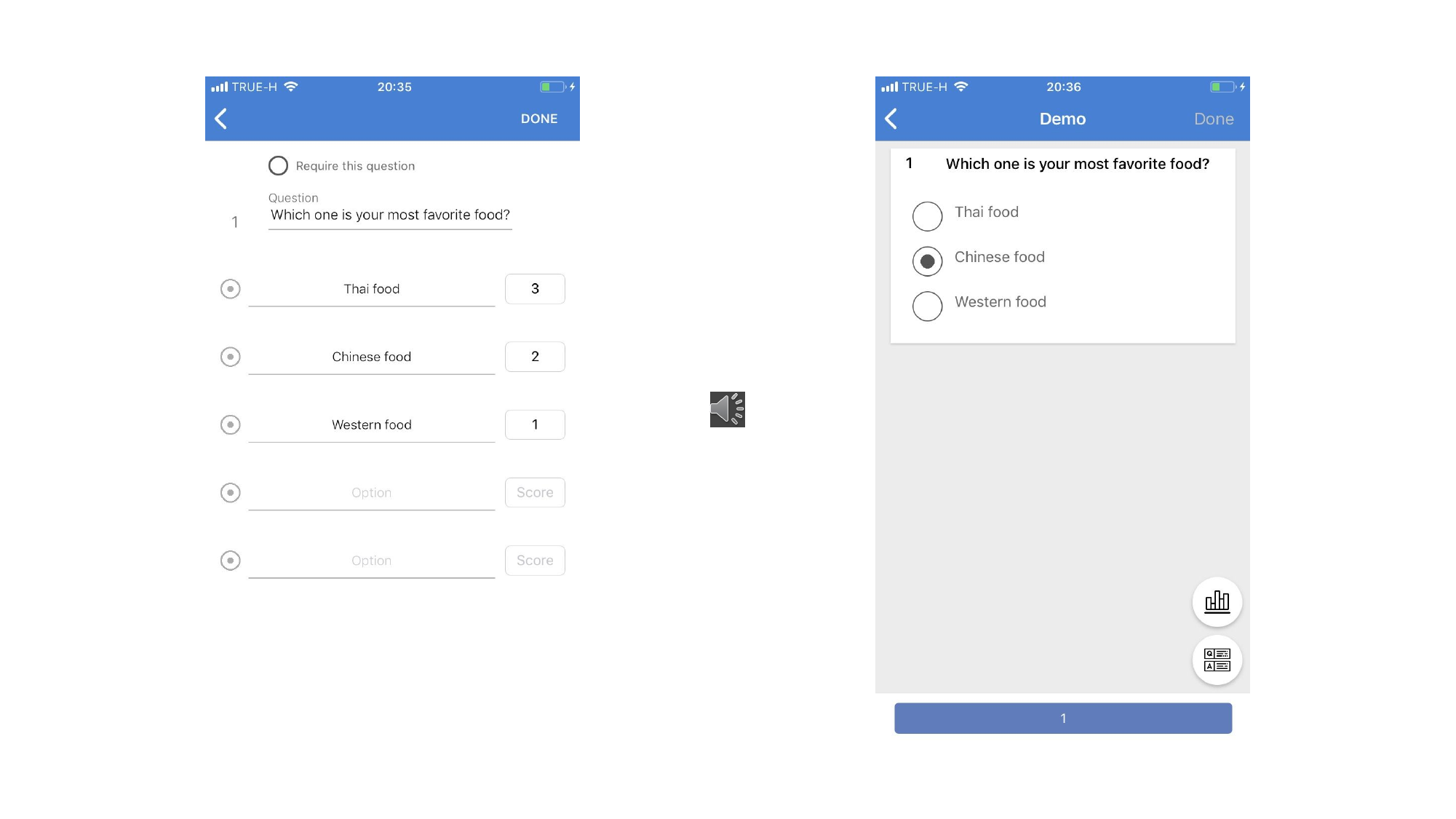

## Slide 34
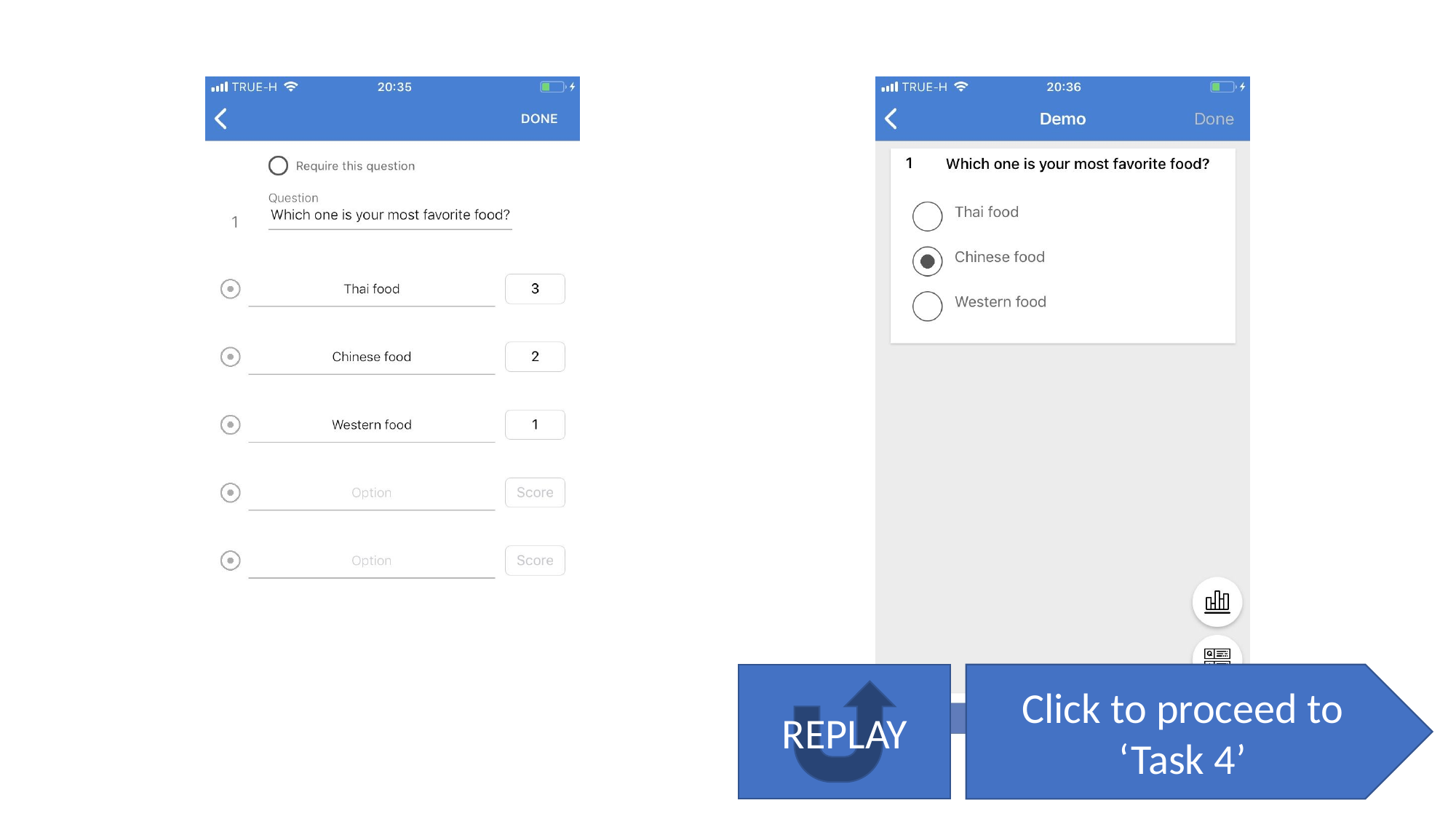

## Slide 35
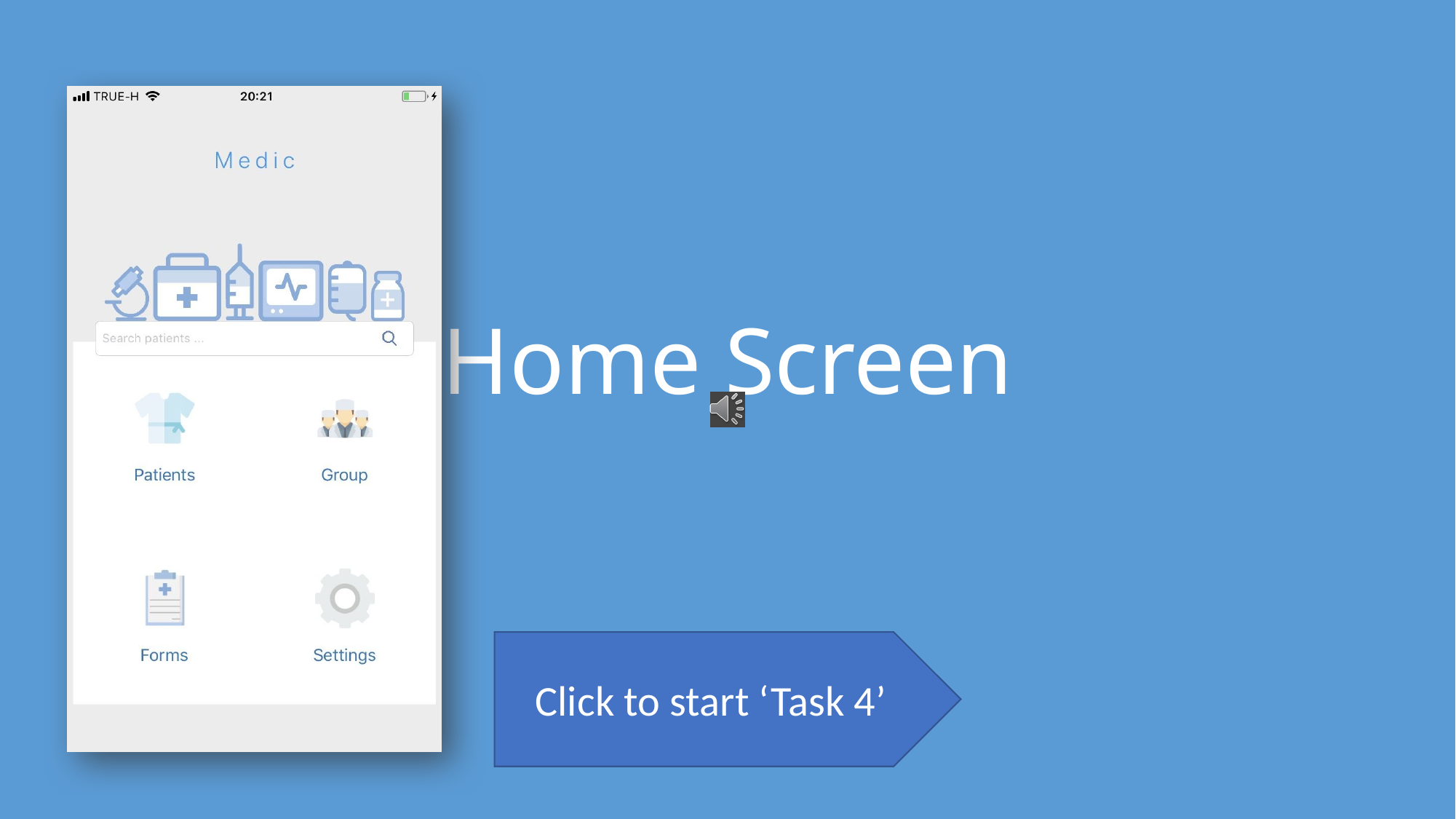

## Slide 36
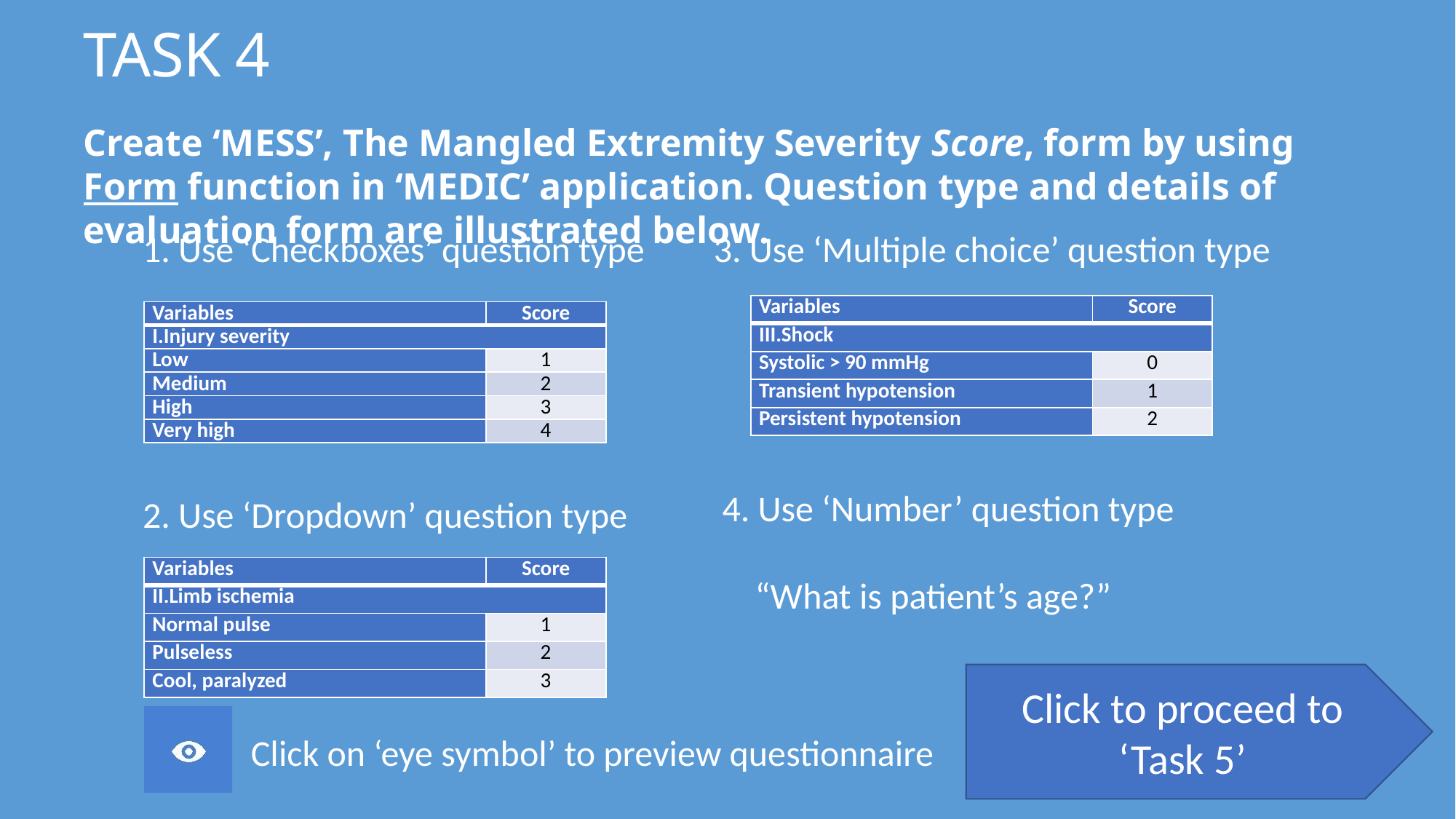

## Slide 37
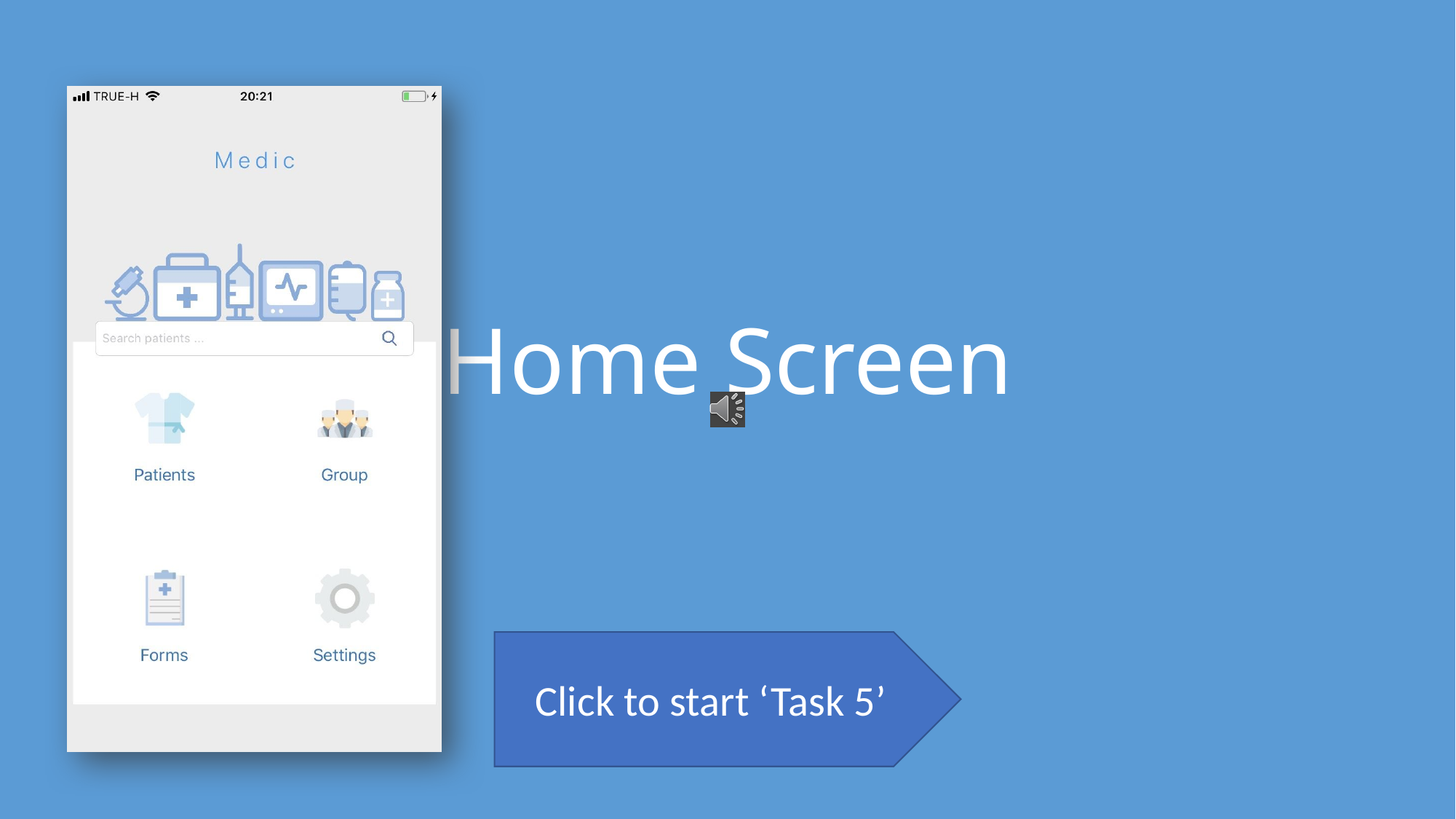

## Slide 38
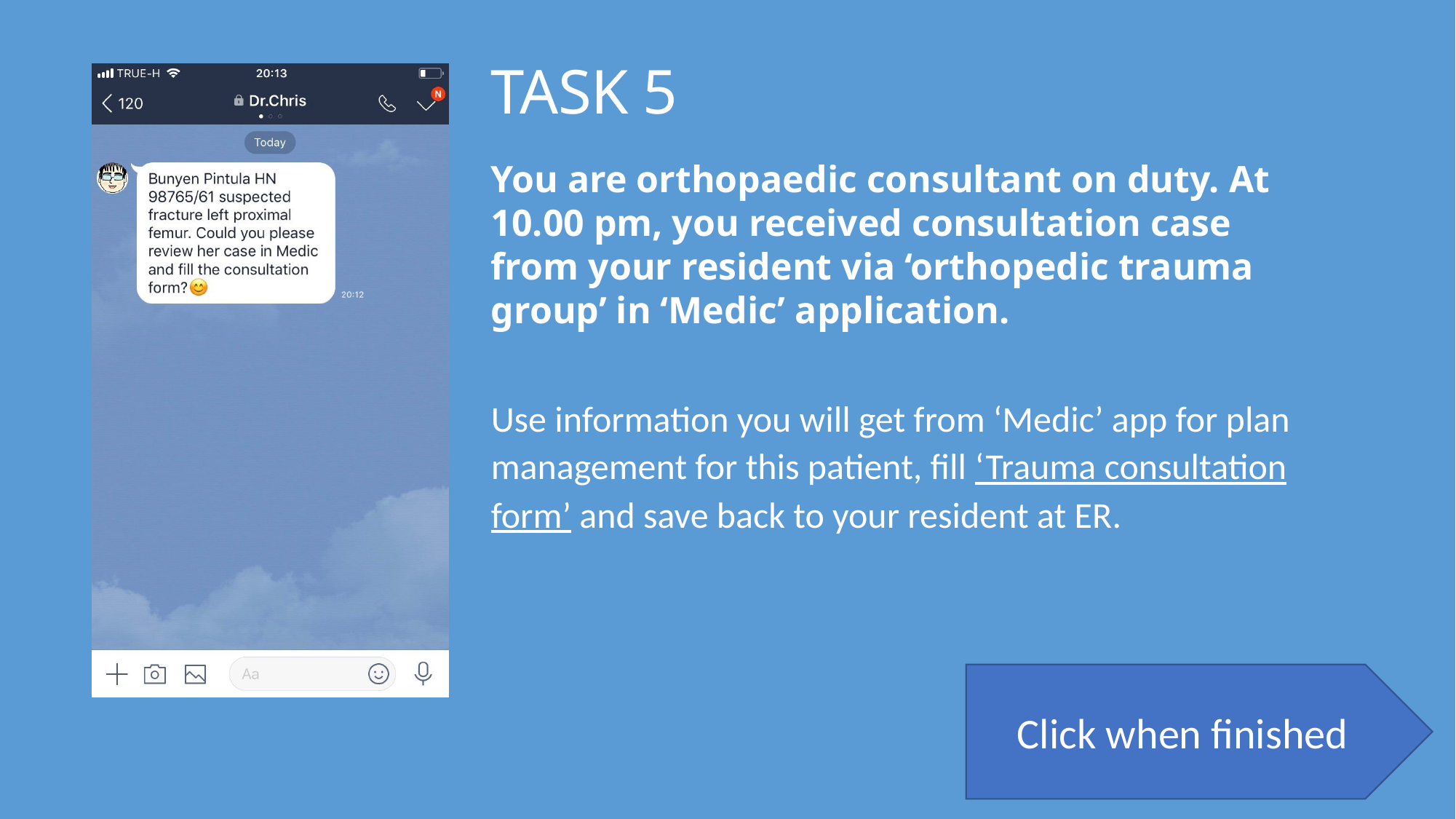

## Slide 39
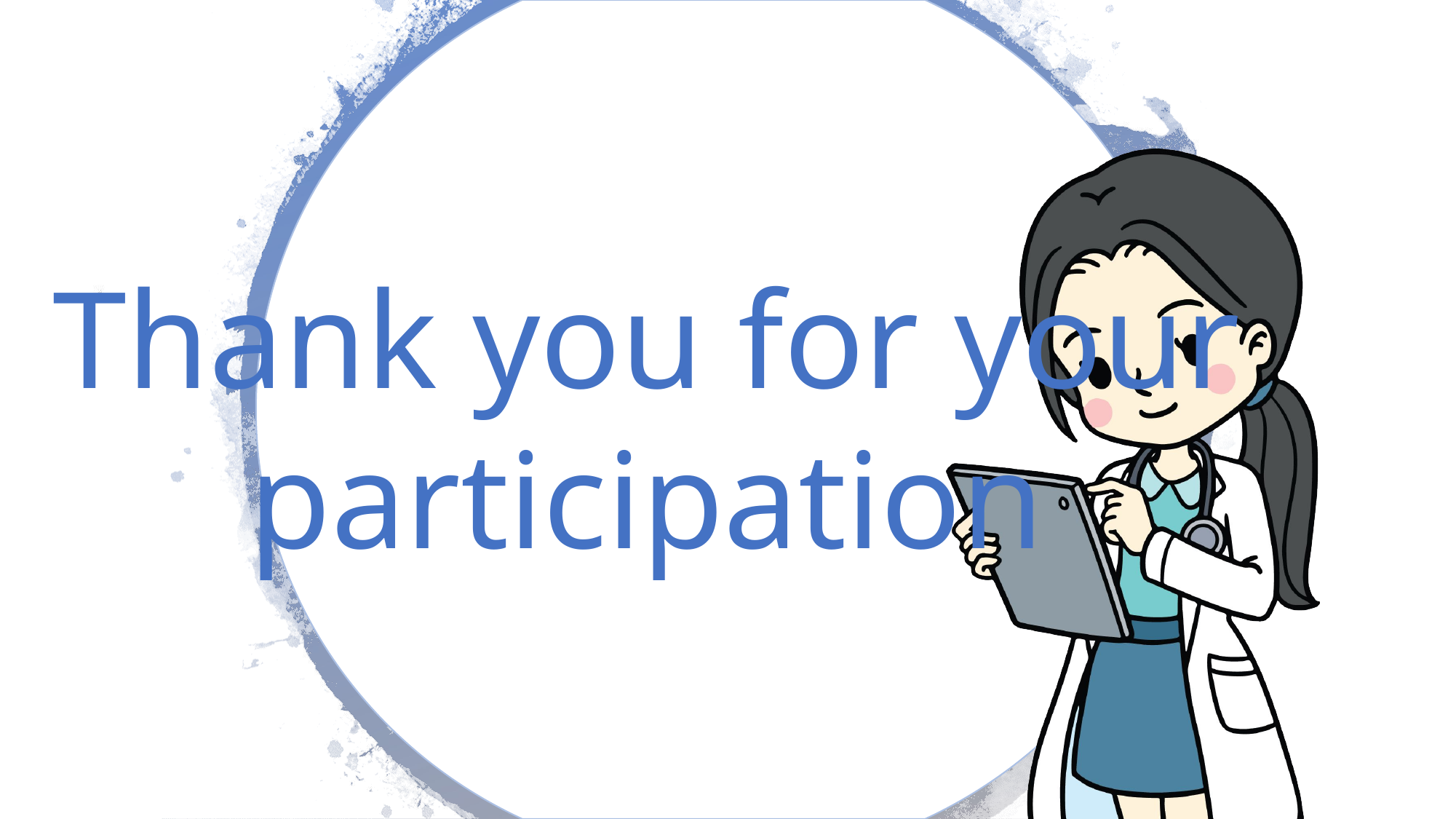

Supplement: Multimedia Appendix 1 [file humanfactors_v8i4e31130_app1.pptx]
